# A smart coating with integrated physical antimicrobial and strain-mapping functionalities for orthopedic implants

**DOI:** 10.1126/sciadv.adg7397

**Published:** 2023-05-05

**Authors:** Yi Zhang, Jinsong Cui, Kuan-Yu Chen, Shanny Hsuan Kuo, Jaishree Sharma, Rimsha Bhatta, Zheng Liu, Austin Ellis-Mohr, Fufei An, Jiahui Li, Qian Chen, Kari D. Foss, Hua Wang, Yumeng Li, Annette M. McCoy, Gee W. Lau, Qing Cao

**Affiliations:** ^1^ Department of Materials Science and Engineering, University of Illinois Urbana-Champaign, Urbana, IL 61801, USA.; ^2^ Department of Pathobiology, University of Illinois Urbana-Champaign, Urbana, IL 61802, USA.; ^3^ Department of Industrial and Enterprise Systems Engineering, University of Illinois Urbana-Champaign, Urbana, IL 61801, USA.; ^4^ Department of Veterinary Clinical Medicine, University of Illinois Urbana-Champaign. Urbana, IL 61802, USA.; ^5^ Veterinary Teaching Hospital, University of Illinois Urbana-Champaign, Urbana, IL 61802, USA.; ^6^ Department of Electrical and Computer Engineering, University of Illinois Urbana-Champaign, Urbana, IL 61801, USA.; ^7^ Department of Chemistry, University of Illinois Urbana-Champaign, Urbana, IL 61801, USA.; ^8^ Frederick Seitz Materials Research Laboratory, University of Illinois Urbana-Champaign, Urbana, IL 61801, USA.; ^9^ Holonyak Micro and Nanotechnology Laboratory, University of Illinois Urbana-Champaign, Urbana, IL 61801, USA.

## Abstract

The prevalence of orthopedic implants is increasing with an aging population. These patients are vulnerable to risks from periprosthetic infections and instrument failures. Here, we present a dual-functional smart polymer foil coating compatible with commercial orthopedic implants to address both septic and aseptic failures. Its outer surface features optimum bioinspired mechano-bactericidal nanostructures, capable of killing a wide spectrum of attached pathogens through a physical process to reduce the risk of bacterial infection, without directly releasing any chemicals or harming mammalian cells. On its inner surface in contact with the implant, an array of strain gauges with multiplexing transistors, built on single-crystalline silicon nanomembranes, is incorporated to map the strain experienced by the implant with high sensitivity and spatial resolution, providing information about bone-implant biomechanics for early diagnosis to minimize the probability of catastrophic instrument failures. Their multimodal functionalities, performance, biocompatibility, and stability are authenticated in sheep posterolateral fusion model and rodent implant infection model.

## Teaser

Smart-coating foils for orthopedic implants prevent infection and provide early diagnosis of instrument failures.

## INTRODUCTION

Orthopedic implants comprise nearly half of the medical implants in use, and their demand has been increasing rapidly with the rising prevalence of orthopedic conditions in the general population, due to demographic changes (*1*, *2*). Despite their widespread use, orthopedic implants continue to pose risks to patients. Periprosthetic infections, which affect 1 to 10% of patients receiving orthopedic implant surgery, are the most common reason for early failure of orthopedics (*3*–*5*). Early diagnosis of implant infections is challenging, and effective treatment typically requires implant removal, followed by surgical debridement of infected tissues (*6*–*8*). Modifying the implant surface to prevent bacterial adhesion (*9*–*11*) or applying surface layers of antibiotics to kill local bacterial populations has been considered for infection risk reduction (*12*–*14*), but they are rarely practiced because of some intrinsic limitations. Coating implant surfaces with hydrophilic molecules or quorum-sensing inhibitors can obstruct bacterial adhesion, but this strategy becomes ineffective once a few bacteria manage to attach, which will eventually develop into an antibiotic-resistant biofilm (*15*). The local delivery of antimicrobial agents from antibiotic-impregnated vehicles loaded on the implant surface is only effective in the short term due to limited reservoir capacity; further, this approach incurs risks of antibiotic resistance, host tissue toxicity, and acute inflammatory responses (*16*–*20*).

Aseptic implant failures are another common complication requiring surgical revisions, affecting over 10% of patients and manifested mainly as loosening caused by mechanical shielding or implant breakage due to fatigue failure (*21*, *22*). These complications are currently diagnosed using x-ray or bone scan imaging. However, radiographic changes can only be detected with gross implant movement, which happens long after the inception of loosening or implant-related microfractures. They also have high cost, only achieve limited sensitivity and specificity, and expose patients to harmful radiation (*23*, *24*). Compared to indirect radiographic signs, direct and real-time monitoring of implant strain can provide useful indicators for early diagnosis and even allow active tracing of dangerous overloads during the postoperative period, which can guide prevention and early intervention to reduce the risk of implant failures. Moreover, the direct and time-series information regarding the biomechanics of the implant-bone system, where the load is dynamically balanced between the implant and bone tissues in different stages of healing, enables clinicians to derive the temporal progression of physiological conditions, such as mechanical shielding, bony fusion, callus formation, and osseointegration, for more patient-specific care to further mitigate risks and improve clinical outcomes. There were attempts to integrate mechanical gauges with orthopedic implants and surrounding tissues (*25*–*27*), but they have not become part of clinical practice due to technological limitations. First, since strain on orthopedic implants under normal physiological loading conditions is small, its accurate detection requires sensors with large gauge factor built on crystalline semiconductors, whose rigid and brittle form factor necessitates their housing within hallow cavities inside implants. The creation of these cavities is technically challenging and economically expensive, and it jeopardizes the mechanical reliability of the implants (*25*, *28*). Second, because of the lack of strategies for multiplexed addressing of individually diced and packaged strain gauges, only single or, at most, a few point measurements can be provided. These limited surface coverage and spatial resolution complicate meaningful clinical judgements, with patients’ individual differences and different surgical procedures leading to spatially complex strain profiles of the implants.

**Fig. 1.**
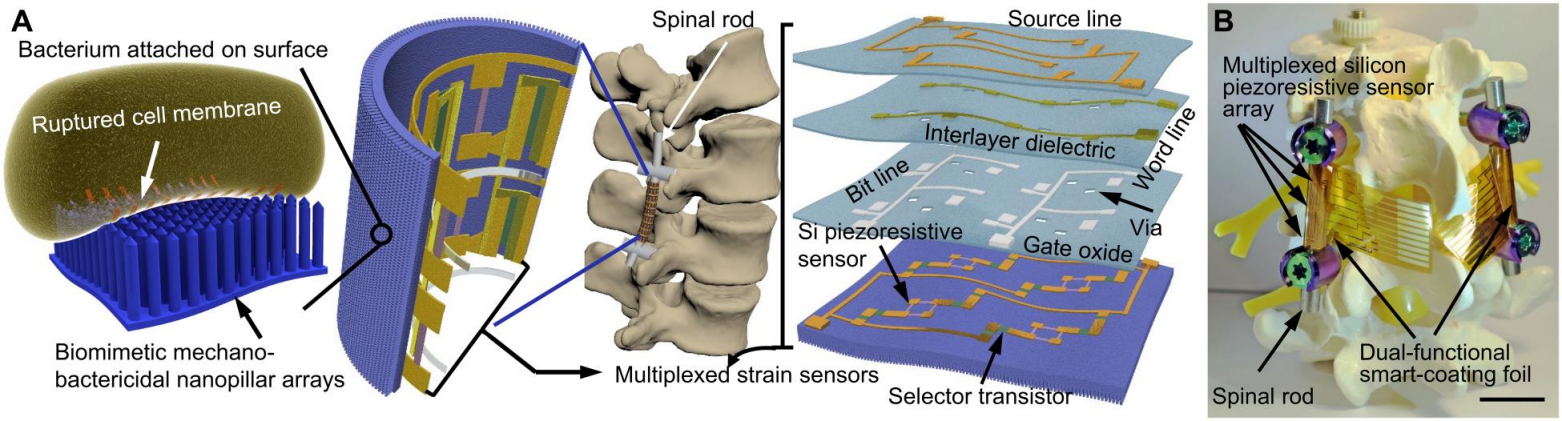
Design of the dual-functional smart-coating foil for orthopedic implants. Schematics (**A**) and optical image (**B**) showing the smart-coating foil with integrated biomimetic mechano-bactericidal and multiplexed strain-sensing functionalities for orthopedic implants (e.g., spinal rods for lumbar fusion). Scale bar, 20 mm.

To simultaneously address these long-standing and critical clinical and technological barriers, here, we present the design of a smart-coating foil that can be conformally applied around the curved surfaces of commercial orthopedic implants and provide both long-term physical bactericidal and sensitive, high–spatial resolution strain-mapping functionalities to mitigate both septic and aseptic orthopedic failures in a soft, integrated form factor (Fig. 1). Its outer surface features arrays of high-density and high–aspect ratio nanopillars with precisely controlled and optimized geometries, mimicking the surface nanotopology of cicada wings (*29*). These biomimetic nanostructures exhibit strong mechano-bactericidal effects against pathogenic bacteria most commonly associated with orthopedic implant infections without directly releasing any chemicals and can effectively prevent their biofilm formation and infection in an in vivo rodent implant infection model. A multiplexed strain-sensing array is built on single-crystalline silicon nanomembranes transfer-printed onto the other side of the foil. The large piezoresistive coefficient of single-crystalline silicon ensures sufficient sensitivity to precisely determine strain down to 0.01% experienced by orthopedic implants, and the nanometer-scale film thickness allows mechanical flexibility for conformal and circumferential surface bonding with commercial orthopedic implants (*30*), e.g., a spinal rod in lumbar fusion. The monolithic integration of silicon gauges with multiplexing transistors in active matrix enables strain mapping across the implant surface to assess associated implant biomechanics, allowing the accurate detection of both early-stage spinal fusion and pedicle-screw loosening as verified in an ex vivo sheep posterolateral fusion model to guide patient-specific care and early intervention against aseptic instrument failures. The strain-mapping array and mechano-bactericidal nanostructures both exhibited excellent biocompatibility and long-term stability in vivo, without causing adversarial cytotoxicity, inducing acute inflammatory responses, or experiencing functional degradation during at least 8 weeks after implantation.

## RESULTS

### Biomimetic physical antimicrobial nanopillar arrays

**Fig. 2.**
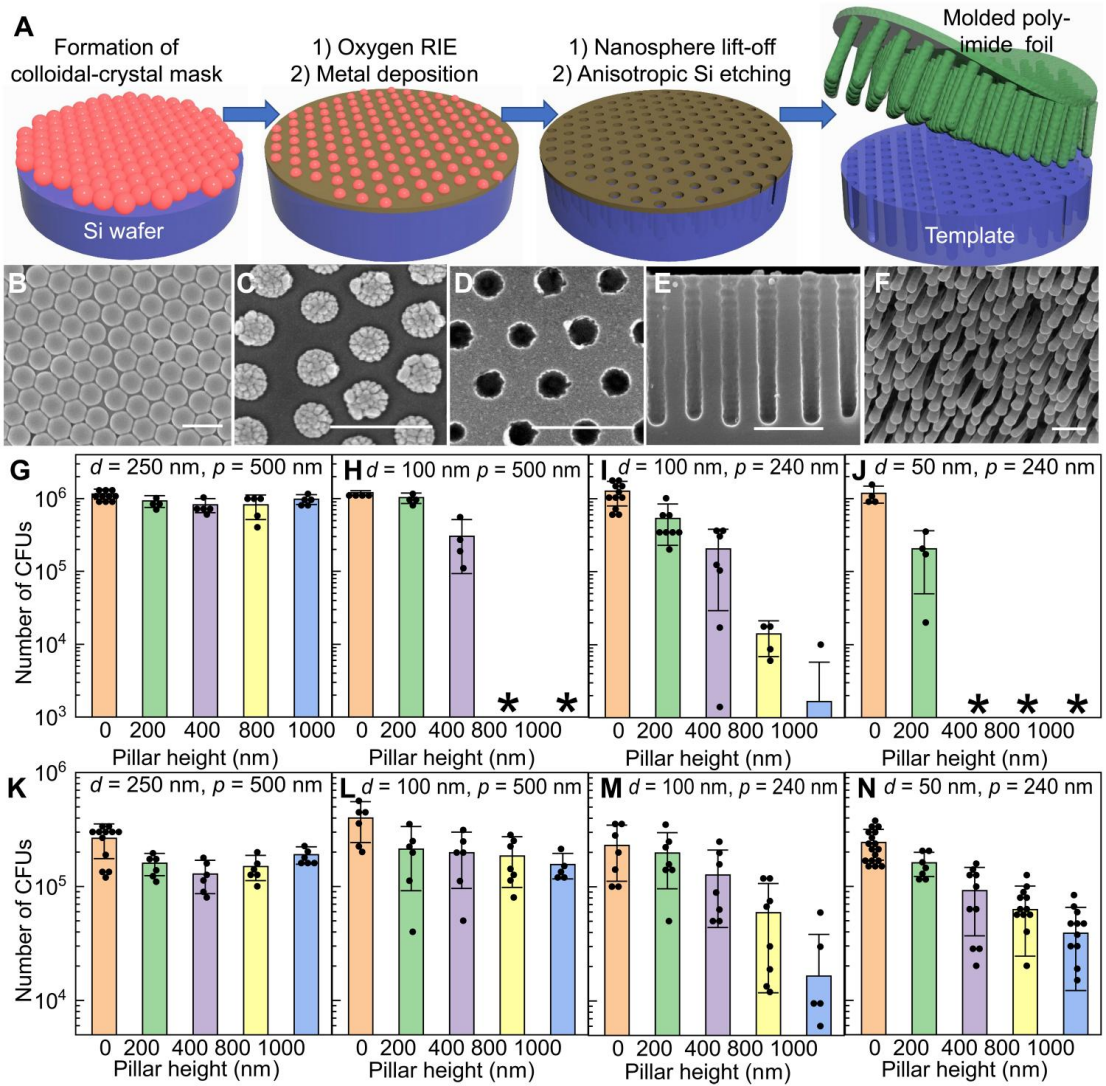
Biomimetic mechano-bactericidal nanopillar arrays on the outer surface of the dual-functional smart-coating foil protecting orthopedic implants against bacterial infections. (**A**) Schematics depicting the process to prepare polyimide foils featuring nanopillar arrays with tunable structures. (**B** to **F**) Scanning electron microscopy (SEM) images showing the critical steps: Top-view SEMs of the colloidal-crystal mask before (B) and after (C) oxygen RIE, and the metal hard mask formed after lift-off (D); cross-sectional SEM micrograph of the template after deep-silicon RIE (E); and tilted-view SEM showing the representative nanopillar arrays on the prepared polyimide foil (F). Scale bars, 400 nm. (**G** to **J**) Counts of viable *E. coli* (MG1655) after being incubated with 1-cm by 1-cm nanopillar arrays with varying nanopillar diameter (*d*), pitch (*p*), and height (*h*) and planar controls for 90 min. Asterisk indicates the number of colony-forming units (CFUs) was less than 1000. (**K** to **N**) Counts of viable MRSA (USA300) after being incubated with 1-cm^2^ nanopillar arrays and planar controls. *p* = 500 nm and *d* = 250 nm for (G) and (K); *p* = 500 nm and *d* = 100 nm for (H) and (L); *p* = 240 nm and *d* = 100 nm for (I) and (M); and *p* = 240 nm and *d* = 50 nm for (J) and (N). Zero height represents the planar foils as internal controls included in each experiment. *N* ≥ 4 in all experiments.

The first key element in the dual-functional smart-coating foil design is the biomimetic mechano-bactericidal nanopillar arrays defined on a flexible polymer substrate. Nanopillar arrays mimicking the nanoprotrusions found on the wing surface of some insects can kill microbes upon contact through deformation and penetration of cell membranes (see text S1 and fig. S1) (*29*, *31*). These mechano-bactericidal nanopillar arrays are more effective in actively killing pathogenic bacteria to prevent their biofilm formation compared to passive antiadhesive surfaces, and they do not directly release any chemicals, which can potentially incur drug resistance or toxic side effects, an essential advantage compared to antibiotics coatings. However, although the nanopillar topology has been indicated as critical to bactericidal efficacy and spectrum in both experiment and simulation (*29*, *32*), it is poorly controlled in existing processes to fabricate synthetic mechano-bactericidal nanotopographies on substrates including silicon wafers, titanium plates, and especially polymer foils, which severely limits their performance and the practical value of this bioinspired antimicrobial strategy (*33*–*38*). Here, we developed a high-throughput process (schematic shown in Fig. 2A and see Materials and Methods for details) combining top-down and bottom-up nanofabrication to prepare large-area, high–aspect ratio polymer-nanopillar arrays with precisely adjustable geometries, which enabled us to achieve and justify the optimum biomimetic design. The process starts by creating a wafer-scale colloidal-crystal mask from monodispersed polystyrene nanospheres (Fig. 2B). An oxygen plasma etching trims down the size of nanospheres and creates uniform interstitial spacings (Fig. 2C). A metal film is then blanketly deposited, followed by the removal of the nanospheres to define a perforated mask (Fig. 2D) used in the subsequent deep-silicon reaction-ion etching (RIE) to create high–aspect ratio wells with vertical sidewalls (Fig. 2E). After coating polyamic acid onto this template, vacuum annealing converts the oligomers into cross-linked polyimide that can be peeled off from the template as a standalone flexible foil, featuring high-density nanopillar arrays on its surface (Fig. 2F). In this process, the nanopillar pitch, diameter, and height are independently and precisely controlled by adjusting the polystyrene diameter, the oxygen RIE time, and the number of etching cycles in the deep-silicon RIE, respectively (see fig. S2 showing 16 different nanopillar-array topologies).

Bactericidal efficacies of prepared nanopillar arrays with systematically varied topologies were quantitatively assessed in vitro against both *Escherichia coli* (strain MG1655) and methicillin-resistant *Staphylococcus aureus* (MRSA; strain USA300), two representative Gram-negative and Gram-positive causative agents of orthopedic implant infections (*6*, *39*), as the first step to determine the optimum biomimetic design (see Materials and Methods). For *E. coli* (Fig. 2, G to J), while keeping the pillar pitch and height identical, nanopillar arrays with smaller diameters killed a substantially larger proportion of bacteria (comparing Fig. 2, G with H, and Fig. 2, I with J), within a short incubation time of 90 min (purposely chosen to ensure the difference in bactericidal efficacy can be determined). These results agree with theoretical models predicting that nanopillars with sharper tips cause larger strains on cell envelope upon contact, leading to enhanced bacterial rupture and death (*32*). In addition to diameter, the pillar height was found to be another critical parameter. Taller pillars induced more severe cell-envelope deformation with the stress-induced deflection of nanopillars (*40*) and, thus, exhibited markedly higher antimicrobial activity (Fig. 2, H to J), without affecting the surface wettability that could influence the bacterial attachment (fig. S3). For *S. aureus*, a similar trend regarding the pillar height and diameter was observed (Fig. 2, M and N). However, there was a major difference between organisms regarding the influence of nanopillar pitch. Reducing the pitch by half, i.e., from 500 to 240 nm, had minimal impact on nanopillars’ killing efficacy against *E. coli* (Fig. 2, H and I) but resulted in markedly enhanced antimicrobial activity against *S. aureus* (Fig. 2, L and M). This difference could be correlated with the difference in microbe size. *E. coli* are about 1 μm by 2 μm on average, and, thus, they are suspended on top of the nanopillar arrays even with pitch as large as 500 nm; while *S. aureus* are smaller with a spherical diameter down to 500 nm, some of them can interstitially adhere between deflected 500-nm-pitch nanopillars to minimize the cell deformation, resulting in higher average cell viability. Smaller pitch also increases the number of contact points, augmenting the chances of local tip rupture to damage the cell envelope, which typically exhibits spatially nonuniform mechanical properties (*32*).

**Fig. 3.**
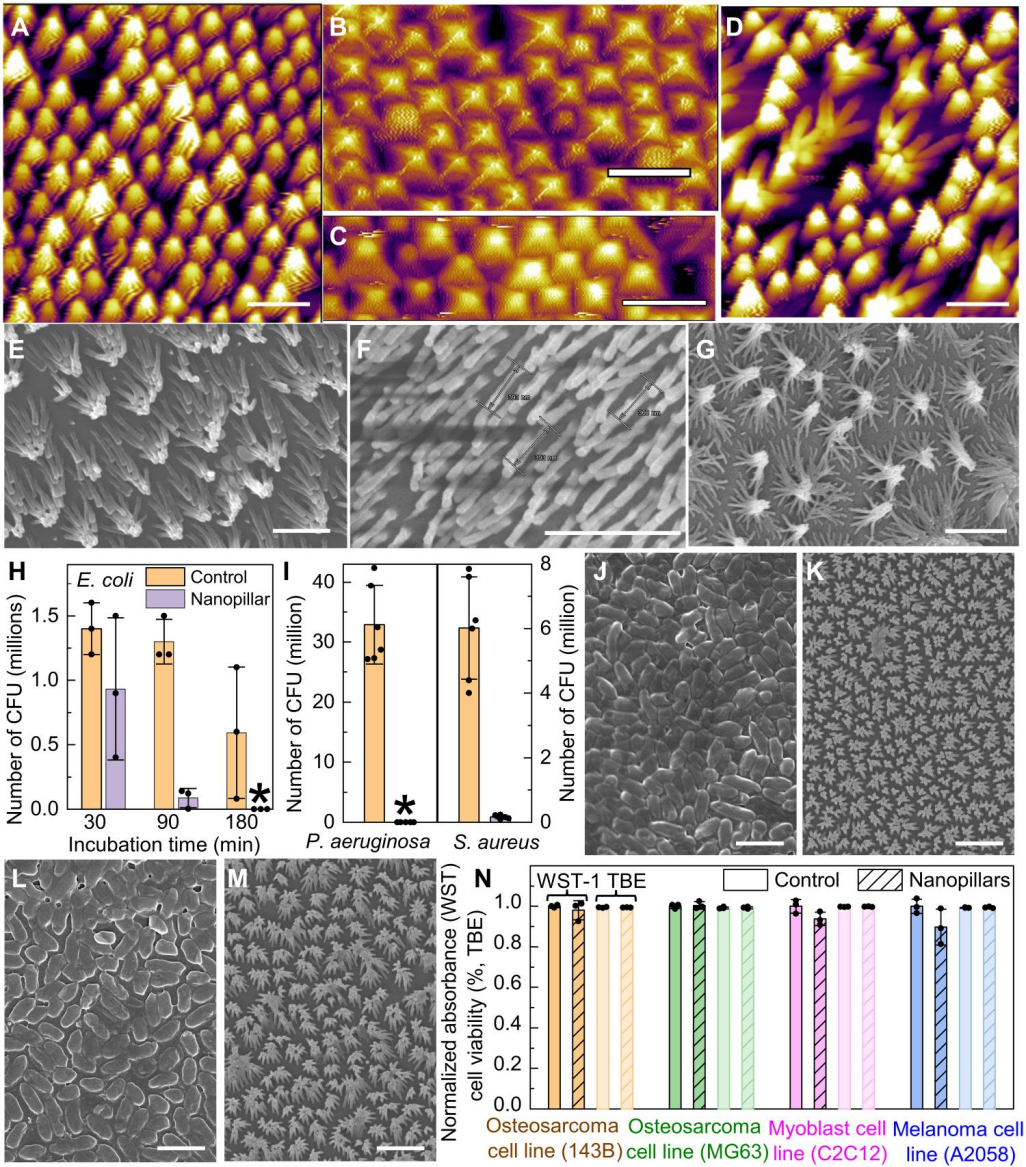
The optimum biomimetic nanopillar-array design ensuring high antimicrobial activities, structural robustness, and biocompatibility with mammalian cells. (**A**) AFM image of freshly prepared mechano-bactericidal polymer nanopillar arrays. (**B** and **C**) AFM scans of 800-nm-tall and 100-nm-diameter polymer nanopillar arrays immersed in water for the first time [(B) *p* = 500 nm as in part A; (C) *p* = 240 nm]. (**D**) AFM image of the nanopillar arrays shown in (A) and (B) after drying. (**E** to **G**) SEM micrographs showing the nanopillar arrays with different geometries after drying [(E) as in (C); (F) *p* = 240 nm, *d* = 100 nm, and *h* = 400 nm; (G) *p* = 240 nm, *d* = 50 nm, and *h* = 400 nm]. Scale bars, 1 μm. (**H** and **I**) The number of CFUs in the suspension after incubation with the planar controls (orange) and nanopillar arrays with the optimum geometry (purple) for *E. coli* strain MG1655 [(H) variable incubation time up to 3 hours, *N* = 3], *S. aureus* strain 29213, and *P. aeruginosa* strain 27853 [(I) incubation for 3 hours, *N* = 6]. (**J** to **M**) SEM showing the bacterial biofilms formed by *S. aureus* on planar (J) and nanostructured (K) foils and *P. aeruginosa* on planar (L) and nanostructured (M) foils, all after 48-hour incubation. The clustering of nanopillars was caused by the cell fixation protocol. Scale bars, 2 μm. (**N**) Mammalian cell viability on polymer nanopillar arrays (hatched) and planar controls (solid) after 48-hour incubation as determined in WST-1 (dark) and TBE (light) assays (*N* = 3). *P* values for unpaired *t* test between control and mechano-bactericidal foils are 0.5, 0.7, 0.08, and 0.1 in WST-1 assay and 0.7, 0.9, 0.5, and 0.5 in TBE test for human osteosarcoma cell 143B, MG63, melanoma cell C2C12, and mouse myoblast cell A2058, respectively.

Although mechano-bactericidal efficacy is enhanced by sharper and taller nanopillars, with smaller (<500 nm) pitch to ensure wide bactericidal spectrum, achieving optimum antimicrobial performance also demands sufficient structural robustness in the usage environment. For 100-nm-diameter nanopillars with a height of 800 nm or above, although they kept their vertically aligned structure when first immersed in water as verified by liquid-phase atomic force microscopy (AFM; Fig. 3, A to C), once they were dried afterward, the lateral capillary force drove them to collapse into cohered pillar clusters with markedly reduced mechano-bactericidal efficacy, regardless of the nanopillar pitch (Fig. 3, D and E) (*41*). In contrast, the 100-nm-diameter, 400-nm-tall nanopillar arrays were able to maintain their structural integrity after washing-drying cycles typically encountered in clinical procedures (Fig. 3F), since halving the pillar height increased the elastic restoring force against the capillary bending force by eight times (see text S2). Regarding the nanopillar diameters, reducing them from 100 to 50 nm results in approximately 2 times smaller capillary bending force but, at the same time, 16 times weaker elastic restoring force. Consequently, although the sharper nanopillars with diameter less than 100 nm can achieve higher initial bactericidal efficacy, they showed severe clustering behavior after drying, similar as the tall (>400 nm in height) pillars (Fig. 3G). We therefore conclude that the optimum topology for mechano-bactericidal nanopillar arrays built on polyimide is ~100 nm in diameter, ~400 nm in height, and ~240 nm in pitch, which quantitatively matches the structures found on cicada wings as favored by evolution (*29*).

This justified optimum biomimetic design ensures a highly effective and wide-spectrum antimicrobial surface. With an area of 1 cm by 1 cm and sufficiently long incubation time of 3 hours to ensure that most bacteria touch the surface in their Brownian motion, it eliminated virtually all viable *E. coli* and *Pseudomonas aeruginosa* [American Type Culture Collection (ATCC), 27853, sepsis clinical isolate], the two most common Gram-negative microorganisms implicated in orthopedic-implant infections (*39*), as well as ~99% of viable *S. aureus*, which comprises up to two-thirds of all pathogens in nosocomial infections (*6*), compared to planar foils as internal controls (Fig. 3, H and I). This high bactericidal efficacy enabled successful prevention of bacterial biofilm formation after 48-hour incubation in a nutrient-rich environment (Fig. 3, J to M, and Materials and Methods). When applying the smart-coating foils featuring the optimum mechano-bactericidal nanopillar design on the curved surfaces of orthopedic implants, the strain will only slightly change the nanopillar pitch and, thus, has negligible impact on their antimicrobial performance, as verified in experiment (fig. S4).

Despite their wide-spectrum and high antimicrobial efficacy, these polymer nanopillar arrays were nontoxic to mammalian cell lines derived from bone, skin, and muscle, including human osteosarcoma cell 143B, MG63, human melanoma cell C2C12, and mouse myoblast cell A2058, after 48-hour incubations, as determined by the standard water-soluble tetrazolium salt (WST-1) assay, which characterized the cell proliferation, viability, and cytotoxicity based on the measured cell metabolic activity, and the Trypan blue exclusion (TBE) test, which directly counted the ratio of total live/total cells (live and dead) to determine the cell survival (Fig. 3N). All treatment groups (planar control and nanopillar arrays) show cell viability at values greater than 98%, which is sufficient for healthy long-phase cultures, with no overt evidence of cell toxicity or accelerated cell death toward the smart-coating foils. Several factors could contribute to this difference. First, mammalian cells and bacteria have quite different envelope and lipid compositions (*42*). As a result, their membranes have different fluidity and, thus, capability to accommodate the imposed surface stress. Second, bacteria maintain 10 to 100 times higher transenvelope osmotic pressure compared to mammalian cells, creating critical mechanical challenge for them to undergo cell envelope remodeling on nanopillar surface. Third, compared to bacteria, mammalian cells contain cytoskeleton systems, which are a dynamic structure composed of actin and tubulin and are capable of transferring external stress to internal structures and organelles to better preserve the cell mechanical integrity. The prepared polymer nanopillar arrays were mechanically robust enough to pass Scotch tape tests and exhibited excellent spatial uniformity, in terms of both structure and quantitative bactericidal efficacy, over a 2.5-inch (6.4 cm) square substrate, which is sufficient to cover the entire surface of implants, e.g., blade plates and spinal rods (fig. S5).

### Multiplexed strain-sensing arrays built on flexible single-crystalline Si nanomembranes

The other essential function realized in the smart-coating foil design is the accurate mapping of local strains on orthopedic implants. Semiconductor piezoresistive sensor arrays, which are built on single-crystalline silicon nanomembranes cultivated from silicon-on-insulator (SOI) wafers and transfer-printed onto the flat side of the Kapton foil substrates opposite to the biomimetic antimicrobial nanopillars (see Materials and Methods and fig. S6), can measure strain down to 0.01% under low operation voltage of 1 to 5 V with good precision and spatial resolution, outside of the scope of capabilities of flexible metal foil strain gauges. These single-crystalline silicon nanomembranes lose their rigidity with sub–100 nm in thickness but preserve excellent intrinsic electrical properties, especially high piezoresistive coefficient and carrier mobility, which enable both sensitive strain gauges and high-performance multiplexing circuits monolithically integrated on the same mechanically flexible substrate (*30*).

**Fig. 4.**
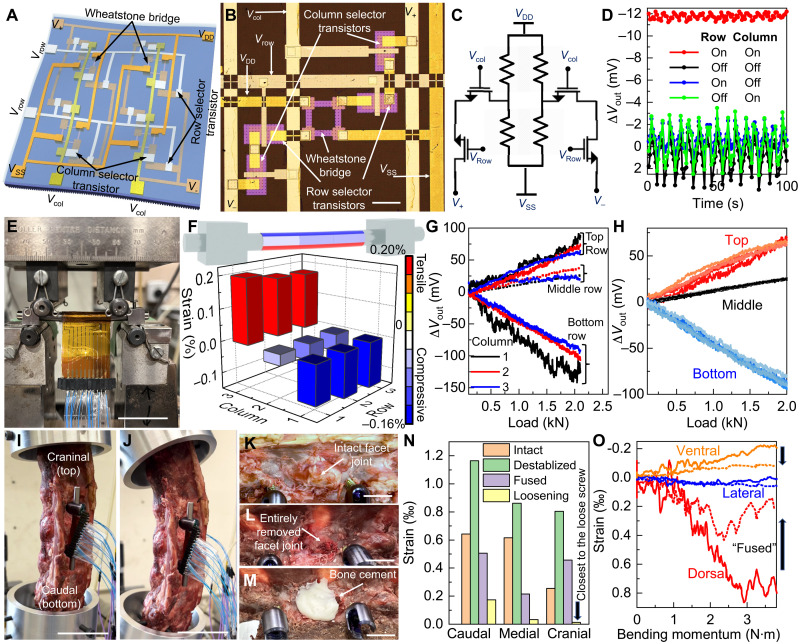
Multiplexed strain-sensing array on the inner surface of the dual-functional smart-coating foil in contact with the orthopedic implants. (**A**) Schematic of the multiplexed strain-sensing array. *V*_DD_, supply voltage; *V*_SS_, ground voltage; *V*_+_, plus-node output-sensing voltage; *V*_−_, negative-node output-sensing voltage; *V*_col_, column-selection voltage; *V*_row_, row-selection voltage. (**B** and **C**) Image [(B) scale bar, 100 μm] and circuit diagram (C) of a single strain-sensing pixel. (**D**) Output voltage difference (∆*V*_out_) of a pixel under applied tensile strain of 0.05%, measured with different bias conditions of the selector transistors. Applied *V*_DD_ = 1 V. (**E**) Optical image of a spinal rod coated with the smart-coating foil under four-point bending test. Scale bar, 50 mm. (**F**) Strain distribution along the spinal rod under 2-kN load as recorded by a 3 × 3 sensor array. (**G**) ∆*V*_out_ recorded by each pixel inside the 3 × 3 array with increasing load applied. Applied *V*_DD_ = 5 V. (**H**) ∆*V*_out_ recorded by the three strain-sensing pixels in the central column during five consecutive loading cycles. (**I** and **J**) Images showing the spine cadaver mounted on the custom platform, without (I) or with (J) the bending moment applied. Scale bars, 50 mm. (**K** to **M**) Images showing the spinal specimen with the intact joint facets (K), after destabilization (L), and after applying bone cement (M). Scale bars, 10 mm. (**N**) Comparing the strain recorded by the strain-sensing pixels located on the dorsal side of the spinal rod for the spinal specimen with intact (orange), destabilized (green), and cemented (purple) facet joint and after pedicle screw loosening (yellow). (**O**) Strain on the spinal rod as measured by the pixels in the medial column of the array, located on the ventral (orange), lateral (blue), and dorsal (red) side of the implant, for the destabilized (solid) and then cemented (dashed lines) spinal specimen.

More specifically, the strain-sensing array is composed of single-crystalline silicon gauges in the Wheatstone bridge configuration, which enhances the sensitivity with differential measurements (*43*), and active row/column selector transistors for multiplexing (Fig. 4A). In each pixel, the outputs of the piezoresistive bridge are routed first to the four selector transistors corresponding to the columns and rows of the active matrix and then to the pads (*V*_+_ and *V*_−_) for voltage sensing (Fig. 4, B and C). Therefore, the pixel output will contribute to the measured voltage difference only with both the row and column selector transistors turned on by positive gate voltage (Fig. 4D). Multiplexing the transistor column/row gate lines and gathering the voltage bias between *V*_+_ and *V*_−_ sequentially create a spatial mapping of the strain distribution. The area footprint of each pixel is about 500 μm by 500 μm, which sets the limit of the spatial resolution in the current design. The adoption of the fast-switching single-crystalline silicon field-effect transistors as selectors enables high refreshing rate, and their large on/off ratio ensures low cross-talk among sensing elements for better accuracy (*44*). Four layers of parylene/Al_2_O_3_/HfO_2_/Al_2_O_3_ stacks are used as passivation on top for isolating electronics from physiological fluids, which can successfully protect the devices in accelerated reliability test [>30 days in phosphate-buffered saline (PBS) buffer at 80°C corresponding to mean time to failure of years (*45*, *46*); fig. S7]. Both HfO_2_ and Al_2_O_3_ are biocompatible and have good long-term stability under physiological conditions (*47*, *48*). There might be some Hf and Al ions released through hydrolysis from the deposited thin films. The Al ions released from these nanometer-thick alumina films will be negligible compared to the background Al ion concentration throughout the human body, while the released trace amount of Hf ions is very unlikely to cause any adversary effect, considering their low toxicity (*49*).

Figure 4E shows an optical image of a smart-coating foil containing a 3 × 3 multiplexed sensor array composed of 36 selector transistors and 36 piezoresistors, which was conformally coated around a commercial spinal rod (ArcasUltra Titanium rod, ArteMedics, St. Paul, MN, USA) subject to strain applied using a four-point bending fixature. Compared to a passive matrix that needs at least 20 input/output lines to connect to 18 high-resolution analog-to-digital converters (ADCs) in the data logger, the active matrix multiplexing of Wheatstone bridge strain sensors markedly reduced the total number of interconnect lines by half and the number of required ADCs to two for more efficient implementation of the whole system for strain mapping, although the incorporation of selector transistors increased the fabrication complexity. A thin layer of spin-casted thermally curable polyurethane (Norland, NEA121) together with a double-sided, medical adhesive tape (3M, 1513) tightly bonded the foil to the implant and mediated the strain transfer. The pixels were located around the circumference and along the axis of the spinal rod, with 4 and 8 mm in pitch along the transvers and the longitudinal directions, respectively. The differences in local strains were accurately captured by this multiplexed array without off-chip amplification (Fig. 4F), showing good agreement with what is predicted in theory (see text S3). The load-strain curves measured by all nine pixels verified that the strain, which increased linearly with the increase in the applied load, was uniform along the axis of the rod but varied from compressive to tensile from the top to bottom of the rod under four-point bending (Fig. 4G). Readings from five consecutive loading-unloading cycles showed good reproducibility without hysteresis (Fig. 4H), with markedly improved signal fidelity compared to what is possible using the commercial metal-foil strain gauges (see text S4 and fig. S8).

The capability of the smart-coating foils equipped with multiplexed silicon strain sensor arrays to determine the progress of spinal fusion and detect implant loosening was then authenticated in an ex vivo sheep posterolateral fusion model (*50*). A radiographically normal spine was harvested from a 1-year-old mixed-breed female sheep. The spine was removed en bloc from T14 to L5, with the epaxial musculature removed but the dorsal spinous ligaments left intact. Pedicle screws (4.5 mm by 30 mm) were placed bilaterally into the vertebral bodies of L2 and L3 to fix 5.5-mm-diameter titanium spinal rods covered with dual-functional smart-coating foils. After removing the transverse processes from L1 and L5 with an osteotome, the specimen was anchored to a custom mechanical testing apparatus affixed to a servo-hydraulic testing machine system (Fig. 4I). Bending moment up to 4 N·m was applied to load the specimen in flexion, simulating physiological movements of the spine (Fig. 4J). Destabilization of the L2 to L3 segment was then performed by removing the articular facets with rongeurs in their entirety bilaterally to simulate the degenerative or traumatic changes to the facet joints (Fig. 4, K and L). After destabilization, the strain on the spinal rod increased by 0.04 ± 0.01% (*P* = 0.04) under the identical bending moment of ~3.8 N·m (Fig. 4N), which verifies that the transfer of more load to the spinal rod, caused by the loss of vertebral stiffness, can be precisely captured by the highly sensitive single-crystalline silicon gauges. The early-to-intermediate stages of fusion were subsequently simulated through filling the facet site with polymethylmethacrylate (Simplex P Bone Cement, Stryker, Kalamazoo, MI, USA; Fig. 4M), whose modulus (~3 GPa) is about 10 times higher than that of facet joints but 5 times lower than that of vertebral bones (*51*). After applying the bone cement to strengthen the interface and thus restore the spinal stiffness, the strain on the spinal rod decreased by 0.06 ± 0.02% (*P* = 0.03), which is consistent with the biomechanical modeling (fig. S9 and text S5). The strain change was most obvious for sensors placed away from the neutral plane, showing the importance of strain mapping to capture the largest strain modulation for more reliable diagnosis (Fig. 4O). After slightly loosening the top pedicle screw by one full turn (fig. S10), the strain on the implant dropped markedly up to 97% (*P* = 0.01), as the implant was mechanically decoupled from the bending vertebrae from one end, with the spatial strain-decreasing pattern pointing to the location of the loose screw (Fig. 4N). These results confirm that the strain distribution change on the spinal rod, which can be accurately measured by the smart-coating foils with sufficient spatial resolution, provides objective biomechanical evidence regarding both the lumber fusion and the implant loosening for the accurate and early diagnosis of pseudarthrosis, which affects 15% of patients receiving spinal fusion surgery (*21*), and aseptic instrument failures. The spatial resolution of the strain mapping can be further increased for more precise and reliable diagnosis by more closely packing these 500-μm by 500-μm size sensing pixels together.

### Functionalities, biocompatibility, and stability of dual-functional smart-coating foils in vivo

**Fig. 5.**
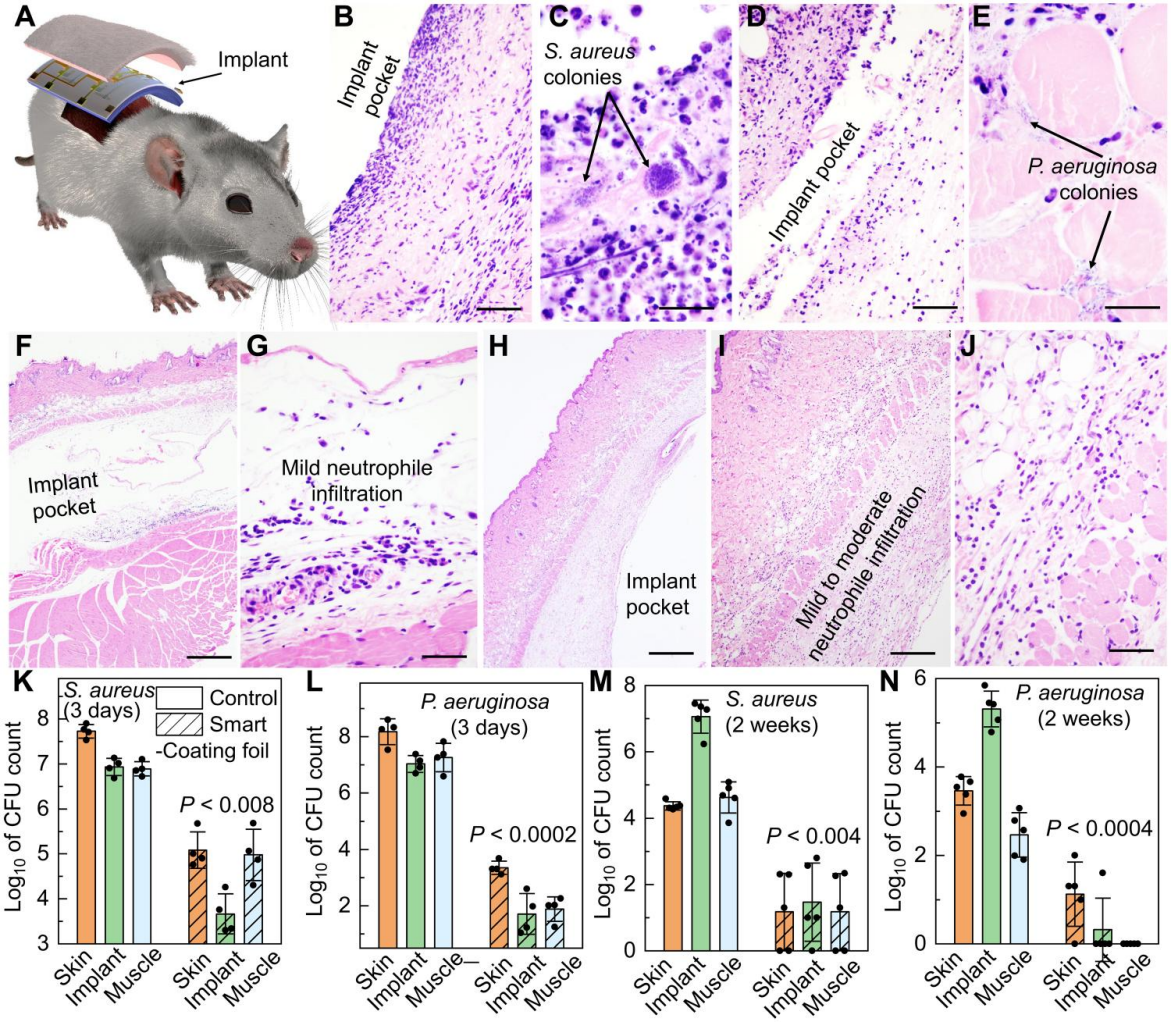
In vivo authentication of antimicrobial performance of the smart-coating foils. (**A**) Schematic showing the mouse subcutaneous implantation model. (**B** to **E**) Images of the hematoxylin and eosin (H&E)–stained histologic sections showing the tissues surrounding the planar foils subject to *S. aureus* (B) or *P. aeruginosa* (D) challenges (scale bars, 50 μm), with the magnified views [(C) for *S. aureus* and (E) for *P. aeruginosa*; scale bars, 20 μm) highlighting the formation of microcolonies. (**F** to **J**) Tissues surrounding the smart-coating foils challenged with *S. aureus* under low [(F) scale bar, 500 μm] and high [(G) scale bar, 50 μm] magnifications or *P. aeruginosa* under low [(H) scale bar, 500 μm], medium [(I) scale bar, 200 μm], and high [(J) scale bar, 50 μm] magnifications, showing only mild neutrophilic inflammation and tissue damage but no evidence of intralesional bacterial colonies. (**K** to **N**) Comparisons of the *S. aureus* (K) and (M) and *P. aeruginosa* (L) and (N) burdens on the implanted planar controls (green, solid bars), the smart-coating foils (diagonal hatched bars), and their surrounding skin (orange) and muscle (blue) tissues after 3 days [(K) and (L) *N* = 4] and 2 weeks [(M) and (N) *N* = 5] in vivo, respectively. *P* values for unpaired *t* test with unequal variance between planar controls and smart-coating foils as well as their associated surrounding tissues are all less than 0.008.

Rodent models were used to authenticate the in vitro results and demonstrated that the smart-coating foils are functional, stable, and well tolerated in vivo. We first performed a bacterial challenge test using the implant infection model to verify the capability of the smart-coating foils to prevent bacterial infections (*52*, *53*). Sterilized 1-cm by 2-cm smart-coating foils featuring mechano-bactericidal nanopillars were first incubated for 4 hours at 37°C with 3 × 10^7^ colony-forming units (CFUs) of either *S. aureus* (strain 29213) or *P. aeruginosa* (strain 27853), both of which are clinical isolates. The number of viable microbes used far exceeded the minimal infective dose, to mimic the surgical site attachment of bacteria on implants from the skin, operating room, or equipment contamination; planar foils served as internal controls. Foil squares were implanted subcutaneously on the back of 7- to 8-week-old mice (four per cohort, equal males and females; Fig. 5A). After 3 days, histological examination revealed that the planar films inoculated with *S. aureus* or *P. aeruginosa* induced fulminant inflammatory cell infiltrates consisting of numerous degenerate neutrophils, fibrin strands, and proteinaceous secretion. Cellular debris and bacterial microcolonies were also prevalent in deep dermis and subcutis, together with glassy hyaline degeneration or necrosis in the underlying skeletal muscle layer (Fig. 5, B to E). In contrast, the mechano-bactericidal nanopillar arrays on the smart-coating foils eliminated intralesional bacterial colonies and substantially reduced the extent of neutrophilic inflammation and tissue damage (Fig. 5, F to J). Quantitatively, the number of CFUs of *S. aureus* and *P. aeruginosa* recoverable from the smart-coating foils and their surrounding tissues was orders of magnitude lower both 3 days (Fig. 5, K and L) and 2 weeks (Fig. 5, M and N) after implantation. Consistent results from a superficial skin infection model further confirmed the in vivo anti-infection effectiveness of the dual-functional smart-coating foil design (fig. S11) (*54*).

**Fig. 6.**
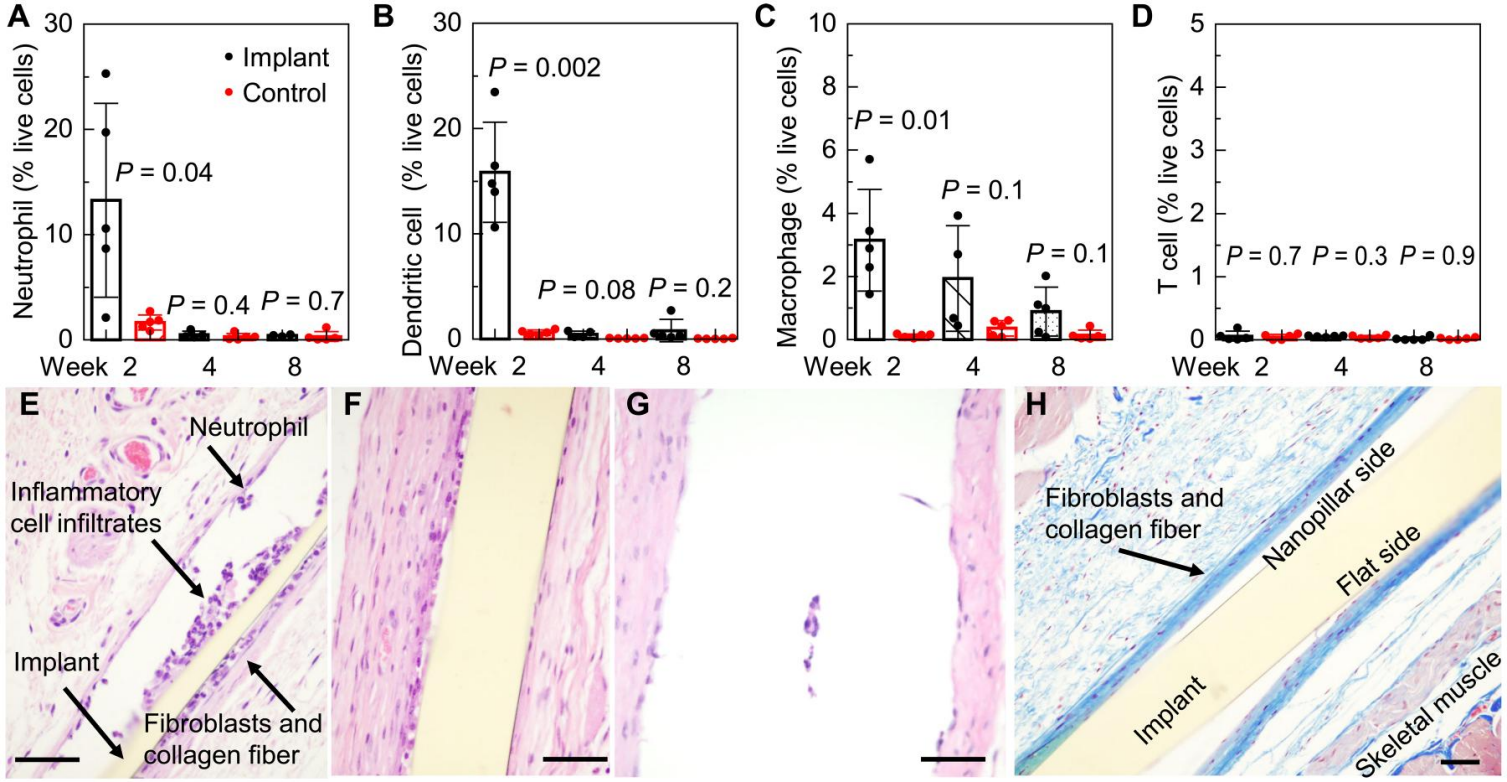
In vivo authentication of biocompatibility of the smart-coating foils. (**A** to **D**) Time evolution of the abundance of neutrophils (A), dendritic cells (B), macrophages (C), and T cells (D) in the tissues surrounding the implanted smart-coating foils (black), with the tissues collected from mice without receiving the surgery as control (red). *N* = 5. *P* values are determined from unpaired *t* test with unequal variance between controls and implants. (**E** to **G**) Histopathology of H&E-stained tissues surrounding the smart-coating foils 2 weeks (E), 4 weeks (F), and 8 weeks (G) (note that the foil was retrieved beforehand for the antimicrobial assays displayed in Fig. 7, D and E) after implantation. (**H**) Masson’s trichrome staining demonstrated a mild degree of peri-implant fibrosis, as evidenced by a thin layer of blue-stained collagen fibers surrounding the smart-coating foil 8 weeks after implantation. Scale bars, 50 μm.

Host inflammatory responses to smart-coating foils were also examined in the mouse subcutaneous implant model. We monitored immune cell populations in axillary, brachial, and inguinal lymph nodes and tissues surrounding the implanted smart-coating foils over 8 weeks (5 mice per cohort). The presence of T lymphocytes, dendritic cells, macrophages, and neutrophils was analyzed by flow cytometry. The results revealed a well-balanced inflammatory response with expected dynamics, indicating no acute adverse inflammatory reactions (*55*). Neutrophils and dendritic cells accumulated at the implant site at week 2, but their numbers quickly returned to baseline, indicating a rapid resolution of the initial inflammatory responses and the progression into the tissue regeneration phase (Fig. 6, A and B). Macrophages, which potentially contribute to the resolution of inflammation and the regeneration of tissues, persisted at the implant-tissue interface with a slower decay, while the difference between control and implant became statistically insignificant (*P* = 0.1 > 0.05) after 4 weeks (Fig. 6C). The absence of T cells suggested the absence of adaptive immune responses throughout the study period (Fig. 6D). Quantitative flow cytometry findings are consistent with histological observations. Hematoxylin and eosin (H&E) staining shows that the numbers of infiltrated neutrophils, macrophages, and foreign body-type multinucleated giant cells all decreased over time (Fig. 6, E to G). Masson’s trichrome staining clearly visualized the presence of thin layers of collagen fibers surrounding the smart-coating foil, with few inflammatory cell infiltrates, neovascularization, and interspersed fibroblasts lying in the collagen fibers, after the foil being implanted for 8 weeks in vivo (Fig. 6H). These results represented a chronic host tissue response, i.e., formation of a protective fibrous layer surrounding the implanted material toward successful implant-tissue integration.

**Fig. 7.**
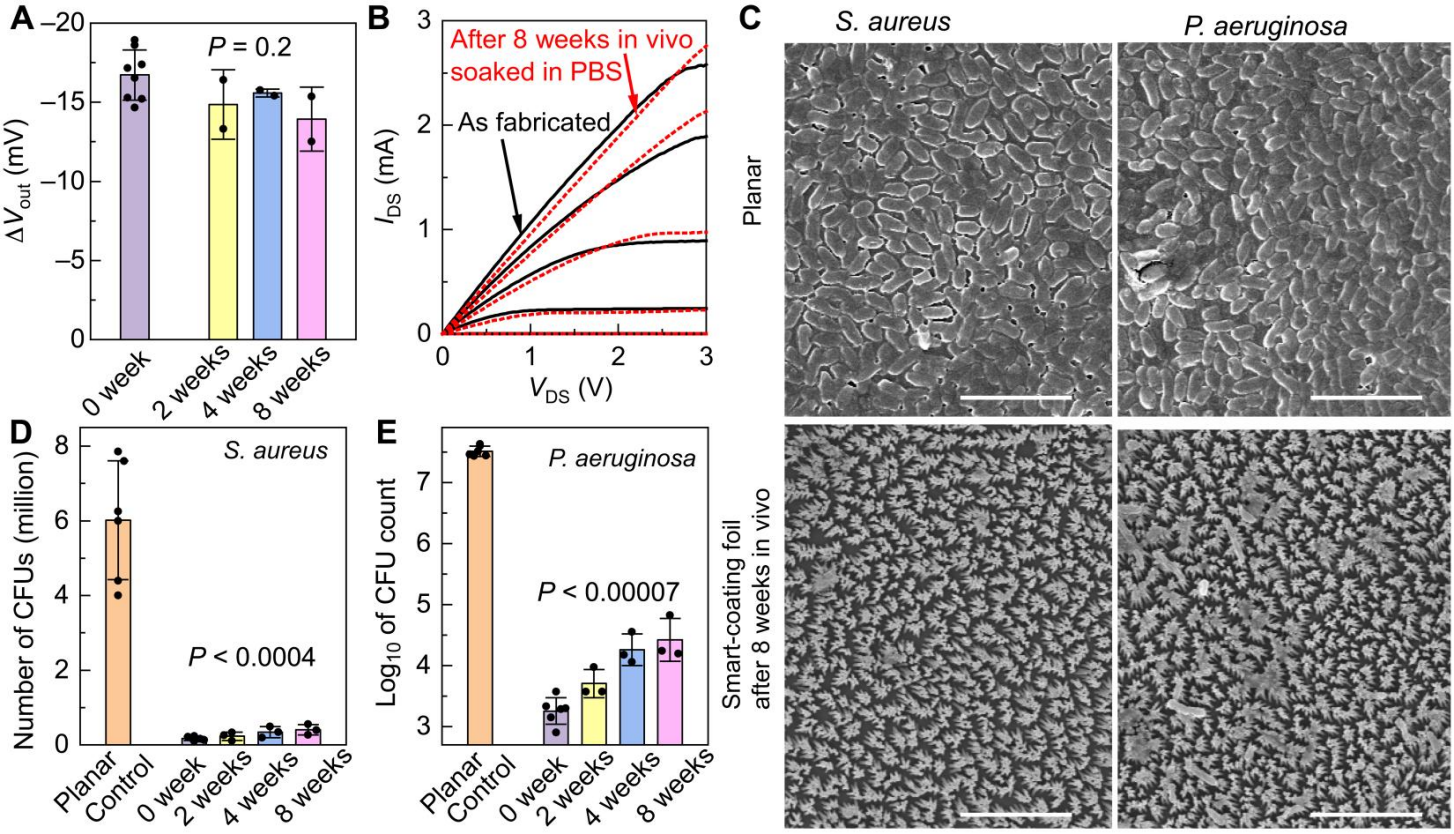
Authentication of long-term stability of the smart-coating foils in vivo. (**A**) ∆*V*_out_ of the silicon-nanomembrane Wheatstone bridge gauges subject to tensile strain of 0.1%, before (purple) and after 2 weeks (yellow), 4 weeks (blue), and 8 weeks (pink) in vivo. *P* value determined by one-way analysis of variance (ANOVA) is 0.2. (**B**) Current-voltage characteristics of the selector transistors before (black solid lines) and after 8 weeks in vivo (red dashed lines), measured with the whole device submerged in PBS. *V*_DS_, source-drain bias; *I*_DS_, source-drain current. (**C**) SEM micrographs showing the in vitro bacterial biofilm formation on the control planar films (top frames) and the mechano-bactericidal smart-coating foils retrieved after 8 weeks in vivo (bottom frames). Films were incubated with 10^5^ to 10^6^ CFUs of *S. aureus* (left frames) or *P. aeruginosa* (right frames) for 48 hours in a nutrient-rich medium. Scale bar, 5 μm. (**D** and **E**) The number of CFUs of *S. aureus* (D) or *P. aeruginosa* (E) after in vitro incubation with the planar controls (orange) or the smart-coating foils that were freshly prepared (0 weeks; purple) or those that were retrieved after subcutaneous implantation in mice for different periods of time, showing their quantitatively similar bactericidal efficacy. *N* ≥ 3. *P* values for unpaired *t* test with unequal variance between planar controls and smart-coating foils are all less than 0.0004.

In addition to the immune compatibility, the mouse subcutaneous implant model also authenticated the long-term antimicrobial and strain-sensing capabilities of smart-coating foils. After 8 weeks in vivo, the strain sensors still produced almost identical ∆*V*_out_ under the same strain applied (Fig. 7A, *P* = 0.2), and the selector transistors exhibited similar current-voltage characteristics, measured with the whole device immersed in PBS (Fig. 7B). These results indicate that the in vivo environment does not degrade either the electronics or the passivation against the penetration of body fluids. The long-term antimicrobial performance of the nanopillar arrays on the other side of the foils was also evaluated. After removing the attached fibrous tissues, the polymer nanostructures were well preserved, and they could still effectively prevent the biofilm formations from either *S. aureus* or *P. aeruginosa* (Fig. 7C). The quantitative bactericidal efficacy was only slightly reduced compared to freshly prepared pristine foils but still maintained >99% bacterial clearance (Fig. 7, D and E).

## DISCUSSION

In summary, we designed and fabricated a smart-coating foil, with optimized biomimetic mechano-bactericidal nanostructures to prevent periprosthetic implant infections and multiplexed strain-sensing systems to continuously monitor the biomechanics of implants for preventive diagnosis against instrument failures, cointegrated in a flexible form factor. Applying the foils on the surface of commercial orthopedic implants (*1*) enabled >99% bacterial clearance in vitro and in vivo across preclinical infection models and (*2*) provided conformal and high spatial resolution active-matrix strain mapping to sensitively detect early-stage bony fusion (*P =* 0.03) and implant loosening (*P =* 0.01) as authenticated in an ex vivo sheep posterolateral fusion model, without modifying the implants’ internal structures. The dual-function integration addresses both septic and aseptic orthopedic failures, enabling improvements in standard of care and patient outcomes. In future explorations, these devices could be valuable for other medical implants, e.g., intravascular catheters and endotracheal tubes, where the prevention of nosocomial infections and the monitoring of mechanical deformation are also critical. The strain-sensing components of the smart-coating foils can be integrated with miniaturized printed circuit boards (PCBs), allowing wireless power and data transfer verified in experiment (fig. S12).

One limitation of our study is that we performed all in vivo studies in mouse models, which do not fully capture the key aspects of host-pathogen interactions and immune responses in human beings. Then, the biomechanical diagnostics was verified in an ex vivo sheep model, which has substantial differences in dimensions compared to human spine, although sheep and human vertebra are most similar in the lumbar region used in this study. Although we can detect significant differences (*P* < 0.05) among healthy, destabilized, and fused spines, as well as significant differences (*P* < 0.01) in cases with implant loosening, further increasing the sensor density can improve the diagnostic reliability. The clinical values of the dual-functional smart-coating foils in orthopedics need further validation in large-animal in vivo models, followed by human trials, which must be implemented on the basis of the fully wireless design without requiring any transcutaneous wires. We therefore recognize the need to further develop the telemetry system to achieve wireless spatial mapping, which can be realized through incorporating commercially available solid-state multiplexer and demultiplexer units on PCBs, with appropriate biocompatible packaging. Additional selector transistors may be required to minimize the power consumption in a battery-free mode of operation for large-scale sensor arrays in vivo (fig. S13).

## MATERIALS AND METHODS

### Study design

The goals of the study were to design and fabricate dual-functional smart-coating foils for orthopedic implants and to validate their performance and functionalities. All protocols were approved by the Institutional Animal Care and Use Committee at the University of Illinois (protocol #20171). Sample sizes were chosen to demonstrate statistical significance by Student’s *t* test. At least triplicates in three independent experiments were performed. We started from evaluating the capability of the smart-coating foils featuring biomimetic nanopillar arrays to kill bacteria and prevent biofilm formation in vitro against *E. coli*, drug-resistant *S. aureus*, and clinically isolated *S. aureus* and *P. aeruginosa*. Their cytotoxicity against human and mouse cell lines derived from bone, skin, and muscle, including human osteosarcoma cell 143B, MG63, human melanoma cell C2C12, and mouse myoblast cell A2058, was also evaluated. To verify their antimicrobial efficacy in vivo, two mouse infection models were tested. In both the implant infection model and the superficial infection model, the outer surface of the smart-coating foils was first incubated with clinically isolated *S. aureus* and *P. aeruginosa* before implantation to mimic the surgical site attachment of bacteria on implants. The effect was analyzed through histopathology, and quantitative bacterial loads were determined on the foil and in the surrounding tissues with both 3 days and 2 weeks as predetermined end points. The induced immune responses in vivo were also analyzed with 8 weeks in vivo as the end point. The strain-mapping function of the smart-coating foils was first evaluated with four-point bending, which measured the gauge factor, linearity, and hysteresis behavior to ascertain the device performance for diagnostic applications. Their capability to detect the small strain modulations under normal physiological conditions corresponding with early-stage bony fusion and pedicle screw loosening was then assessed in an ex vivo sheep posterolateral fusion model. The device reliability and potential degradation were lastly tested in vivo using a mouse subcutaneous implant model, together with ex vivo evaluation of antimicrobial and strain-sensing performances.

### Preparation of the large-area polyimide foils featuring biomimetic mechano-bactericidal nanopillar arrays with precisely tunable geometries

SiO_2_ (90 nm) was grown on a single-crystalline silicon wafer via thermal oxidation. After rendering the SiO_2_ surface hydrophilic with oxygen plasma (Harrick Plasma cleaner, 18 W, 500 mtorr, 10 min), a solution containing 5 wt % of monodispersed polystyrene nanospheres (Thermo Fisher Scientific) suspended in a 400:1 mixture of isopropyl alcohol and Triton X-100 (Sigma-Aldrich) was spin-casted on top (6000 rpm for 30 s) to form a hexagonally close-packed monolayer of nanospheres. The deposited nanospheres were then time-etched in oxygen RIE [Plasma-Therm; under 100-W radio-frequency power with oxygen flow rate of 20 standard cubic centimeters per minute (SCCM) to maintain a chamber pressure of 100 mtorr] to the desired size and spacings. The etching rate was about 100 nm min^−1^. A thin (~10 nm) layer of Cr was then blanketly deposited by electron-beam evaporation (Temescal). After removing the nanospheres with the Cr on top by dissolving the polystyrene in chloroform (Sigma-Aldrich), a perforated Cr mask was formed on the SiO_2_ surface. The pattern was subsequently transferred to the thermal oxide layer by etching the SiO_2_ in the exposed regions in CF_4_ plasma (Plasma-Therm; 20 SCCM CF_4_, 50 mtorr, 300 W). Following the removal of the Cr layer by Cr etchant (Transene), deep-silicon RIE was used to produce nanowell arrays as the template for micromolding. Each cycle in the deep RIE [SPTS Technologies inductively coupled plasma (ICP)] was composed of an etching step where the flow of 130 SCCM of SF_6_ and 13 SCCM of O_2_ was used to maintain a plasma with a coil power of 600 W and a platen power of 12 W for 5 s, followed by a deposition step where the flow of 85 SCCM of C_4_F_8_ helped to maintain a fluorocarbon plasma with a coil power of 600 W and a platen power of 0 W for 3 s. The etching depth per cycle was limited to about 20 nm to produce a nearly vertical sidewall. Removing the SiO_2_ mask by hydrofluoric acid (HF) completed the fabrication of the templates. In the molding process, poly(pyromellitic dianhydride-*co*-4,4′-oxydianiline) amic acid (Sigma-Aldrich) was coated on the master template to a certain thickness as controlled by doctor blading, followed by degassed in vacuum for 12 hours. A 250°C annealing in vacuum (1 torr) for 1 hour subsequently cross-linked the polyamic acid liquid precursors into a solid polyimide film through thermal imidization. A slow ramping rate (3° to 5°C min^−1^) was necessary to promote the diffusion of the polyamic acid precursors into the nanowell with nanometer-size hydraulic radius. After cooling down to room temperature, the polyimide film featuring high-density nanopillar arrays on one surface was peeled off from the master template as a foil, with its top surface remaining flat.

### Fabrication of the multiplexed strain-sensing array on the flat side of the polyimide foils

SiO_2_ (400 nm) was first grown on an SOI wafer (Soitec; 70-nm lightly p-doped device layer silicon on 1-μm buried oxide) by plasma-enhanced chemical vapor deposition [Oxford Plasma Enhanced Chemical Vapor Deposition (PECVD) system; the deposition was completed in 4.5 min under 20 W and a chamber temperature of 380°C, using SiH_4_ flowing at 8.5 SCCM and N_2_O flowing at 710 SCCM as the precursors as well as N_2_ flowing at 162 SCCM as the carrier gas to maintain a chamber pressure of 1000 mtorr]. Photolithography was then performed to define patterns of the heavily doped source-drain regions of the selector transistors and contact regions of the piezoresistive strain sensors into the photoresist (AZ-5214), followed by removing the PECVD SiO_2_ in the exposed areas using buffered oxide etchant with the photoresist as the etching mask. After stripping the photoresist in acetone, a film of phosphorus-containing spin-on-dopant (Filmtronics, P509) was blanketly deposited by spin-casting at 3000 rpm for 30 s, followed by a soft bake at 110°C for 3 min. Annealing at 850°C for 10 min in a three-zone tube furnace (Lindberg) with N_2_ (2 liters min^−1^) and O_2_ (1 liter min^−1^) flow drove the phosphorus to diffuse from the spin-on-dopant into the underlying silicon to form the heavily n-doped contact regions. After cooling down to room temperature, the wafer was immersed in HF to remove both the spin-on-dopant and the thermal oxide mask, followed by piranha cleaning to remove the residual phosphorus oxide. This diffusion-doping process was then repeated to dope the channel of the piezoresistive strain sensors at a reduced annealing temperature of 750°C to achieve a lower doping concentration around 1 × 10^18^ cm^−3^, which minimizes the silicon channel’s temperature coefficient of resistance for better device stability without severely degrading its piezoresistive coefficient. After completing doping, another photolithography was performed to pattern a layer of photoresist into a mesh composed of two-dimensional (2D) arrays of 10-μm by 10-μm square openings with 50 μm pitch. The silicon in the exposed square-opening areas was removed by etching in an ICP RIE system (Oxford; the BCl_3_ flowing at 10 SCCM, together with Ar flowing at 5 SCCM, maintained a chamber pressure of 10 mtorr; the applied ICP power and RIE power were 300 and 100 W, respectively; the etching time was 1 min), following the undercut etching of the buried oxide in concentrated HF to release the silicon nanomembrane from the SOI wafer. A layer of polyvinyl alcohol (PVA; Sigma-Aldrich; 15 wt %) was further spin-casted (2000 rpm for 2 min) on top to provide both mechanical support and surface protection. A thermal release tape (Revalpha) was applied onto the PVA film. Peeling off the thermal release tape picked up the PVA film together with the silicon nanomembrane. The whole film stack was then laminated on the flat side of the polyimide foil covered with a layer of ultraviolet (UV)–cured polyurethane (Norland, NEA 121; spin-casted at 3000 rpm for 60 s and then exposed to UV lamp at intensity of 20 to 25 mW cm^−2^ for 90 s) as the adhesion layer to bond with the silicon nanomembrane. Heating the foil on hotplate at 160°C released the tape, leaving the silicon nanomembrane with the PVA protection layer on the polyimide receiving substrate. The PVA film was lastly removed by immersing the foil in 60°C water bath for 10 min, which completed the silicon nanomembrane transfer-printing process. To fabricate the multiplexed strain sensor arrays on the polyimide foils based on transferred silicon nanomembranes, the channel and contact regions of each selector transistor and strain sensor were first protected by photoresist defined by photolithography, with the silicon in the exposed regions removed by XeF_2_ (Xactix XeF_2_ etcher; 3 torr for 1 min) with high etching selectivity against the polymer substrate, for device isolation. Afterward, the source-drain electrodes of the selector transistors, the contact pads to the strain sensors, and the first level of interconnects were patterned into the photoresist by photolithography, followed by electron-beam evaporation of 2-nm Cr/100-nm Au/0.2-nm Cr and lift-off in acetone. The 0.2-nm Cr was used as the seeding layer for the subsequent growth of the gate oxide/interlayer dielectric on top, which was composed of 40-nm HfO_2_/30-nm Al_2_O_3_ bilayer, by atomic layer deposition (ALD; Savannah 100) at 120°C using tetrakis(dimethylamido)hafnium and triethylaluminum as precursors, respectively, together with water. The low deposition temperature prevented cracking of the oxide layer resulting from the mismatch of the thermal expansion coefficients, and the top Al_2_O_3_ layer helped to seal pinholes for higher device yield. After the dielectrics had been deposited, vias for interlayer interconnects were exposed in photoresist by photolithography, followed by etching of the HfO_2_/Al_2_O_3_ bilayer in ICP-RIE (Oxford; with CHF_3_ flowing at 10 SCCM and Ar flowing at 5 SCCM, the chamber pressure was kept at 5 mtorr; under the RIE power set at 200 W and ICP power at 300 W, the total etching time was 4 min). Two-nanometer Cr/100-nm Au/0.2-nm Cr was then blanketly deposited by sputtering (AJA). The sputtering was used instead of the electron-beam evaporation here to ensure sufficient side-wall coverage of the via holes and the continuity of the interconnect lines despite the underlying surface topography. The gate pattern for the selector transistors and the second-level interconnects were defined with one additional photolithography step and the etch-back scheme, using commercial wet etchants (Transene) to remove gold and chromium in exposed areas. ALD deposition of the 40-nm HfO_2_/30-nm Al_2_O_3_ interlayer dielectrics, the opening of contact vias by ICP-RIE, as well as the sputtering deposition and the etch-back patterning of the interconnect lines were then repeated twice to form two additional metal-interconnect levels on top, which completed the fabrication of the multiplexed sensor array on the flat side of the dual-functional smart-coating foils.

### Multi-layered passivation of the strain-sensing electronics to complete the fabrication of the dual-functional smart-coating foils

After completing the multiplexed sensor arrays, an encapsulation layer, which was composed of five repeating stacks of parylene/Al_2_O_3_/HfO_2_/Al_2_O_3_, was deposited on top to isolate the electronics with the physiological environment and complete the fabrication of the dual-functional smart-coating foils for orthopedic implants. The parylene was deposited using the Specialty Coating Systems parylene coater. The deposition chamber was held at room temperature with chamber pressure of 35 mtorr. The temperature of the vaporizer and the pyrolysis furnace was kept at 175° and 690°C, respectively. The film thickness was controlled to about 200 nm by loading 0.5 g of diparaxylylene (Galentis) as precursor. The HfO_2_ and Al_2_O_3_ were both deposited at 120°C using the aforementioned ALD process. If the strain-mapping electronics were fabricated on a planar Kapton sheet rather than directly on the back side of the nanostructured mechano-bactericidal polyimide film, an additional step was required to bond them together using the thermally curable polyurethane as the adhesion layer.

### Bactericidal efficacy determined by the cell-viability plate counts

Bacteria (*E. coil* strain MG1665, MRSA strain USA300, *S. aureus* strain 29213, and *P. aeruginosa* strain 27853) were first incubated overnight in Luria-Bertani (LB) broth at 37°C and then diluted with PBS (1×). After adjusting to the desirable optical density, 40 μl of bacterial solution containing 10^5^ to 10^7^ cells was added onto the 1-cm by 1-cm area of each test surface and spread out as a thin film. After incubation for different time intervals between 30 to 180 min, bacteria were thoroughly washed from the test surfaces by rinsing with 1 ml of PBS (1×) for multiple times. The collected bacterial washes were serially diluted and then spread onto LB agar plates. After incubation overnight at 37°C, the number of CFUs was determined by visual counting. All experiments involved planar Kapton foils as internal controls and a minimum of quadruplicates from four independent experiments, with the quantitative bactericidal results expressed as means ± SD.

### Biofilm growth conditions and cell-fixation protocol for scanning electron microscopy

*S. aureus* and *P. aeruginosa* were grown in LB broth overnight at 37°C, washed three times in sterile PBS, and diluted to approximately 10^6^ CFUs ml^−1^, in the M63 medium supplemented with magnesium sulfate, glucose, and casamino acids as the carbon and energy source. The diluted cell dispersion (100 μl) was then added onto the 1-cm by 1-cm area of each test surface and incubated in a humidified chamber (styrofoam box containing prewet paper towel) for 48 hours. After incubation, the samples were fixed by immersing the samples in PBS containing 4% (volumetric ratio) paraformaldehyde for 30 min at room temperature. Afterward, the samples were washed in PBS, followed by dehydration in graded ethanol series of 25, 50, 75, 90, and 100% (twice), each time for 15 min. Last, all the samples were air-dried in a desiccator and coated with gold using a sputter coater to prevent charging effect in electron microscopy imaging. All samples were examined by scanning electron microscopy (SEM) to determine the presence of biofilms. Planar Kapton foils were used as internal controls. Consistent results were obtained from triplicates from three independent experiments.

### Mammalian cell cytotoxicity analysis

Smart-coating foils featuring nanopillar arrays and planar controls (*N* = 3 per group) were placed in six-well plates and sterilized by UV exposure for 30 min. Human osteosarcoma 143b cells (1 × 10^6^; ATCC, CRL-8303), human osteosarcoma MG63 cells (ATCC, CRL-1427), mouse skeletal myoblast C2C12 cells (ATCC, CRL-1772), or human melanoma A2058 cells (ATCC, CRL11147) were then seeded into each well for each group, respectively, and incubated first for 6 hours. Afterward, cultured media were changed to fresh media and incubated for additional 48 hours. In the WST-1 assay, the incubated cells were washed by PBS and incubated for another 2 hours in 400 μl of mixture of cell proliferation reagent WST-1 and fresh media (premixed at 1:10 ratio). Reacted mixtures were then transferred to 96-well microtiter plates, and the absorbance was measured against a background control at 460 nm to quantitatively determine the cell proliferation and viability. In the TBE test, the incubated cells and foils were first washed by PBS and trypsinized. An aliquot of cell suspension being tested for viability was then centrifuged at 200*g* for 5 min. After discarding the supernatant, cell pellets were resuspended in 1 ml of PBS containing 0.4% trypan blue (TB) dye. One part cell suspension was incubated for about 3 min at room temperature. One drop of the TB/cell mixture was then applied to a hemacytometer. Quantitative cell viability was calculated as viable cell ratio = total number of viable cells / total number of cells per milliliter of aliquot.

### In vivo bacterial challenge tests in mouse implant infection and superficial infection models

CD-1 mice (7 to 8 weeks old, four to five for each cohort, equal males and females) were first anesthetized by intraperitoneal injection of ketamine (80 to 100 mg kg^−1^) and xylazine (10 to 12.5 mg kg^−1^). For the implant infection model, a 1-cm incision was made through the depilated skin of the dorsum with a #15 blade. The skin was bluntly dissected from the underlying tissue layers using iris scissors, forming a subcutaneous pocket into which the smart-coating foils or planar controls were placed. Before their subcutaneous implantations, both the smart-coating foils and the planar controls had been first sterilized and then incubated for 4 hours at 37°C with (i) 3 × 10^7^ CFUs of *S. aureus* 29213 or (ii) 3 × 10^7^ CFUs of *P. aeruginosa* 27853, in their liquid suspensions to mimic the surgical site attachment of bacteria on implants. The incision was lastly closed with tissue glue. For the superficial infection model, after anesthesia, the fur on the dorsal of mice was first removed by shaving, followed by exfoliating cream. An area of ca. 1 cm by 2 cm was then tape-stripped with Tensoplast, an elastic adhesive bandage, 10 times in succession to disrupt the skin barrier by partial removal of the epidermal layer. The smart-coating foils and planar controls, after sterilization and incubation with *S. aureus* and *P. aeruginosa* as described in the implant infection model, were affixed onto the stripped skin with surgical tapes. Mice were monitored for up to 2 weeks. The smart-coating foils and the planar controls, together with the associated surrounding skin and muscle tissues, were first collected en bloc from all animals for analyzing the histopathological changes and then vortexed for 1 min in 1 ml of sterile saline to collect supernatant to determine the bacterial burdens using the standard plate count technique.

### In vivo biocompatibility and stability assessment

Following the aforementioned surgical procedure, the sterile smart-coating foils were implanted subcutaneously in CD-1 mice. At 2, 4, and 8 weeks after implantation, tissues surrounding the smart-coating foils as well as axillary, brachial, and inguinal lymph nodes were harvested for the analyses of T cells, dendritic cells, macrophages, and neutrophils by flow cytometry. The multiplexed strain-sensing and antimicrobial functionalities of the retrieved smart-coating foils were both characterized in vitro.

### Near-field communication telemetry

The wireless, battery-free operations were realized through harvesting energy from the radio-frequency field to power a commercial near-field communication (NFC) chip (Texas Instruments, RF430FRL152H), which drives the flexible piezoresistive sensors integrated on the smart-coating foil and provides data storage and voltage regulation. The chip includes a microcontroller, both volatile and nonvolatile memory, and three 14-bit analog-digital converters. An NFC reader (TRF7970AEVM) wirelessly delivers power and data using ISO 15693 protocol at 13.56 MHz.

### Instrumentation

Top-view SEM micrographs were acquired using a Hitachi S4800 microscope. Cross-sectional focused ion beam (FIB)–SEM was performed using a FEI Helios 600i dual-beam system. The electron beam with an accelerating voltage of 5 kV was used for imaging. The FIB was performed at 30 kV. An ion beam current of 9.3 nA was used to cut a large window for viewing cross section, and that of 2.5 nA was used for cleaning. AFM topography images of nanopillar arrays were measured using an Asylum Research MFP-3D equipped with a fluid cantilever holder in contact mode, with aluminum-coated silicon AFM probes (Ted Pella, TAP300AL). The mechanical bending tests of the spinal rods and the spine specimen were conducted using an Instron Model 1331 Servo-Hydraulic Frame. The multiplexed strain mapping was acquired using a data acquisition system (NI USB-6251) connected to the sensor arrays via flat flexible cables. Direct current measurements of the individual strain sensors and selector transistors were performed in ambient using a manual probe station connected with a semiconductor parameter analyzer (Keysight, B1500A) equipped with integrated high-resolution source measurement units (Keysight, B1517A). The AZ 5214E photoresist was patterned with a Heidelberg MLA150 aligner.

### Statistical analysis

Data are presented with average values and SD. Statistical analyses were performed using SPSS. Statistically significant differences, which were attributed to a variable with *P* < 0.05, were determined by unpaired and two-tailed Student’s *t* test for cell viability assessment, bacterial burden comparison, and flow cytometry analyses of immune cells. Paired and two-tailed Student’s *t* test compared the strain change in the ex vivo sheep posterolateral fusion model before and after simulated fusion. Two-tailed one-sample Student’s *t* test evaluated the relative strain change after loosening. One-way analysis of variance (ANOVA) was used for determining the stability of strain sensors in vivo. *P* values are included in the appropriate figure legends, figure captions, and main text.

## Supplementary Materials

### This PDF file includes:

Supplementary Text S1 to S5 Figs. S1 to S15 References
