# Supplementary material for: A smart coating with integrated physical antimicrobial and strain-mapping functionalities for orthopedic implants
Source: Sci Adv. 2023 May 5;9(18):eadg7397. doi: 10.1126/sciadv.adg7397 (PMC10162669; doi:10.1126/sciadv.adg7397)
Supplement: Supplementary file 1 — Supplementary Text S1 to S5 Figs. S1 to S15 References [file sciadv.adg7397_sm.pdf]

Science Advances
AAAS

Supplementary Materials for

A smart coating with integrated physical antimicrobial and strain-mapping functionalities for orthopedic implants

Yi Zhang *et al.*

Corresponding author: Qing Cao, qingcao2@illinois.edu

Sci. Adv. **9**, eadg7397 (2023)
DOI: 10.1126/sciadv.adg7397

This PDF file includes:

Supplementary Text S1 to S5
Figs. S1 to S15
References

Supplementary Text

Supplementary Text 1: Mechanisms causing the death of bacteria on biomimetic polymer nanopillar arrays.

There are several mechanisms proposed regarding the mechano-bactericidal effect. Some biophysical models suggest that the nanopillar arrays stretch the region of the cell membrane suspended between neighboring nanopillars beyond its elastic limit, resulting in membrane rupture and bacterial death (*29*). The biomechanical models, one the other hand, indicate that the largest strain, driven by intermolecular adhesion forces, develops at the pillar apex instead of the suspended regions, leading to localized rupture and penetration of the cell envelope (*32*). Meanwhile, recent proteomic analysis revealed that nanopillars induce oxidative stress within bacterial cells as evident from the elevated levels of oxidative-stress proteins and H_2O_2 (*34*).

Here we performed cross-sectional FIB-SEM analysis of the bacteria sitting on top of the bactericidal polymer nanopillar arrays (*40, 56, 57*). The bacteria were first incubated on the biomimetic antimicrobial nanopillar arrays (240 nm pitch, 400 nm height, and 100 nm diameter) for 48 hours. After fixation, the areas with bacterial attachment were first identified in top-view SEM, then iteratively cross-sectioned in FIB, and simultaneously imaged *via* SEM. A 200 nm-thick protective layer of platinum was blanketly deposited on top by sputtering before slicing to minimize the charging effect. The cross-sectional FIB-SEM analysis of a *S. aureus* sitting on top of the polyimide nanopillar arrays was displayed in Fig. S1A and S1B. There were at least four nanopillars in contact with this specific cell (marked as nanopillar #1–4). Three of them (nanopillar #1, 3, and 4) interacted with the side of the *S. aureus*, only causing the deformation of the bacterial envelope morphology through indentation. The other nanopillar (nanopillar #2), on the contrary, had clearly penetrated the bacterial envelope, but without causing the loss of the turgor pressure. For *P. aeruginosa* (Fig. S1C and S1D), at least five neighboring nanopillars were in contact with the cell. The deflection of the nanopillars with bending toward the center of the cell and the penetration through the cell envelope by all nanopillars were both observed. The cell envelope was significantly deformed likely caused by cytoplasmic leakage. The higher probability for nanopillars to pierce through the envelope of Gram-negative bacteria (*P. aeruginosa*) than that of their Gram-positive counterparts (*S. aureus*) which are strengthened by a much thicker peptidoglycan cell wall potentially explains the observed quantitative difference in achieved bactericidal efficacy.

The bacterium-nanopillar interface visualized by FIB-SEM shows that the nanopillars are able to pierce through the bacterial envelope and disrupt the continuous barrier between the cytosol and the extracellular environment, which can cause substantial alterations to the bacterial morphology and even the loss of the turgor pressure. These results suggest that the local penetration of the cell membrane at the pillar apex as the plausible mechanism responsible for the mechano-bactericidal effect of polymer nanopillar arrays. But further investigations are required to confirm, which will be the subject in our future studies.

Supplementary Text 2: Capillary bending and elastic restoring forces of nanopillars.

High-aspect-ratio nanopillar arrays are susceptible to deformation. Especially, as water evaporates off the surface, the nanopillars bend and could potentially cluster together due to lateral capillary menisci interaction forces (*41*). Such capillary force F_c between two neighboring nanopillars partially immersed in a liquid can be calculated as

$$F_c = -\frac{\pi\gamma d^2 \cos^2\theta}{2\sqrt{p^2 - d^2}},$$

where γ is the liquid-vapor interfacial energy; θ is the equilibrium contact angle; d is the nanopillar diameter; and p is the nanopillar pitch. While the capillary force bends the tips of nanopillars toward each other to form clusters, it is counter-balanced with the elastic restoring force F_e:

$$F_e = \frac{3\sqrt{2}\pi E d^4 \delta}{64 h^3},$$

where E is the modulus, δ is the deflection, and h is the pillar height (*58*). The clustering can be avoided as long as that $F_e > F_c$ can be maintained over a reasonable range of deflection δ.

By reducing the nanopillar height h by half from 800 nm to 400 nm while keeping both d and p identical, the elastic restoring force F_e is therefore increased by eight times to significantly improve the structural robustness of nanopillars against deformation as shown in Fig. 3F. On the contrary, by reducing the diameter d by half from 100 nm to 50 nm while keeping both p=240 nm and h identical, although the capillary bending force F_c is reduced approximately by half, the elastic restoring force F_e is drastically reduced by 16 times, leading to the enhanced clustering behavior observed in experiment (Fig. 3G).

Supplementary Text 3: Flexural strain on the spinal rod in four-point-bending test.

Fig. S14 illustrates the spinal rod subject to a four-point-bending test. The diameter (d) of the rod (ArteMedics, Grade 23 Titanium) was 5.5 mm. It was attached to two 9 mm-wide steel cubes with screws to limit the rotation of the rod during measurement, which were placed on two supports that were 60 mm apart (L_{total}). The cylindrical actuators (diameter 10 mm) applied two equal point loads up to 2 kN in total on the spinal rod. The distance (L) between the support and the actuator on the same side was 9.5 mm. With the symmetric geometry of the test step, the fore (F) applied at each contact was 1 kN. The maximum deflection recorded in the center of the rod (δ) was ~0.5 mm.

Based on the classical Euler–Bernoulli beam theory, the maximum stress on the rod (σ) can then be calculated as:

$$\sigma = My/I$$

where M is the moment applied at the middle of the rod defined as:

$$M = F \cdot L,$$

I is the moment of inertia, which can be calculated as

$$I = \frac{\pi (d/2)^4}{4},$$

and y is the distance from the neutral plane of the rod to its utmost fibre, which is the rod radius (d/2).

Meanwhile, the effective modulus (E) can be calculated as

$$E = \frac{FL(3L_{Total}^2 - 4L^2)}{24\delta I}.$$

The maximum flexural strain is then derived as

$$\varepsilon_{\max} = \frac{\sigma}{E} = \frac{\frac{My}{I}}{\frac{FL(3L_{Total}^2 - 4L^2)}{24\delta I}} = \frac{24\delta y}{(3L_{Total}^2 - 4L^2)} = 0.32\%$$

More accurate results were further obtained based on the finite-element simulation utilizing the software package COMSOL. The 3D geometry in the model comprised the Ti rod, the steel support blocks, and the cylindrical actuators. The simulated ε_{max} as a function of the load applied is displayed in Fig. S15A, suggesting ε_{max}=0.25% under 2 kN load, which has a better agreement with the 0.21% maximum tensile strain we measured in experiment. The simulated cross-sectional and lateral strain tensor distributions (Fig. S15B and S15C) quantitatively agree with the experimental strain-mapping results shown in Fig. 4F.

Supplementary Text 4: Strain on the spinal rod measured using the commercial metal-foil strain gauges and off-chip amplifiers.

To further validate the results obtained from the multiplexed single-crystalline silicon piezoresistive sensor arrays and demonstrate their superior performance, we performed the identical four-point bending of the spinal rod with the commercial metal-foil strain gauge (MMF403994, Micro-Measurements) applied on its surface instead (Fig. S8A), as bonded with the same medical-grade polyester tape (3M 1513). The sensor was consisted of a small-section Constantan-alloy filament deposited on a backing plastic substrate. Under applied tensile or compressive strain, the metal wire changes its shape and thus the electrical resistance end-to-end, with a gauge factor of 2. The resistance of the sensor was 120 Ω. During the measurement, it was connected with another three off-chip resistors to form a Wheatstone bridge and the differential voltage signal was amplified with an off-chip amplifier by 1,000×. Since there was no multiplexing capability with the individually packaged commercial sensor, it was sequentially applied to three different locations around the circumference of the spinal rod, as illustrated in Fig. S8B, to record the strain in three separate load cycles, where the load was increased linearly from 0.1 to 2.1 kN with a rate of 20 $N \cdot s^{-1}$. Even after the 1,000× off-chip amplification of the signal and utilizing a much higher drive voltage of 13 V, the data measured using the conventional metal-foil gauge still exhibited much worse signal-to-noise ratio and thus unreliable measurement results compared to what was recorded using the multiplexed single-crystalline silicon piezoresistive sensor arrays, which have ~25 times higher piezoresistive gauge factor and built-in multiplexing for strain mapping, even though the strain measured under the maximum 2 kN load was quantitatively comparable (Fig. S8C).

Supplementary Text 5: Finite-element modeling of the strain distribution on a spinal-rod implant in interbody fusion.

A finite-element model was developed for simulating the biomechanics associated with the posterolateral spinal fusion using COMSOL Multiphysics. The exact geometry of the spinal vertebra in the model was imported from BodyParts3D, a 3D structure database for anatomy (*59*). As shown in Fig. S9A, the model contained two vertebral bodies with Young's modulus of 12 GPa

and an intervertebral spinal disc with a lower modulus of 42.7 MPa (*60, 61*). The backside view of the model shows the facet joints, which were simplified to a cylindrical shape. Their Young's modulus was set to 2.28 MPa for destabilized facet joints (*62*), and 6 GPa for early-to-middle stage fused facet joints. The spinal rod and screws made of titanium, whose modulus was set as 110 GPa (*63*), were affixed to the vertebral bodies without loosening. We then simulated the spinal movements in flexion by applying 150 N compression force to the topper part of the vertebral bodies (Fig. S9B). The strain fields in the axial direction of the spinal rods along their surface were estimated and compared between the spinal models with either destabilized or fused facet joints. The simulated strain on the spinal rod demonstrated a large spatial variation, which reflects the importance of straining mapping. The maximum flexural strain reduced significantly after the simulated fusion of facet joints, which agrees both qualitatively and quantitatively with what we observed in experiment.

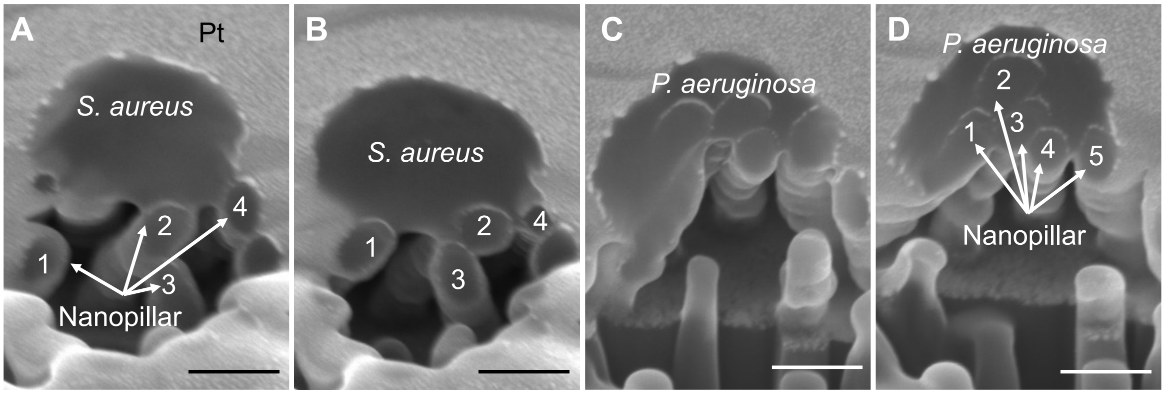

Fig. S1. **Cross-sectional analysis of bacteria on polymer nanopillar-array surface suggesting biomimetic mechano-bactericidal nanopillars penetrating and deforming the cell envelope.** (**A-B**) Analysis of *S. aureus* (strain 29213) cross sections taken at two different locations, showing that nanopillar 2 penetrated the cell envelope. (**C-D**) Analysis of *P. aeruginosa* (strain 27853) cross sections taken at two different locations, showing that all five nanopillars penetrated the cell envelope. Scale bars: 200 nm.

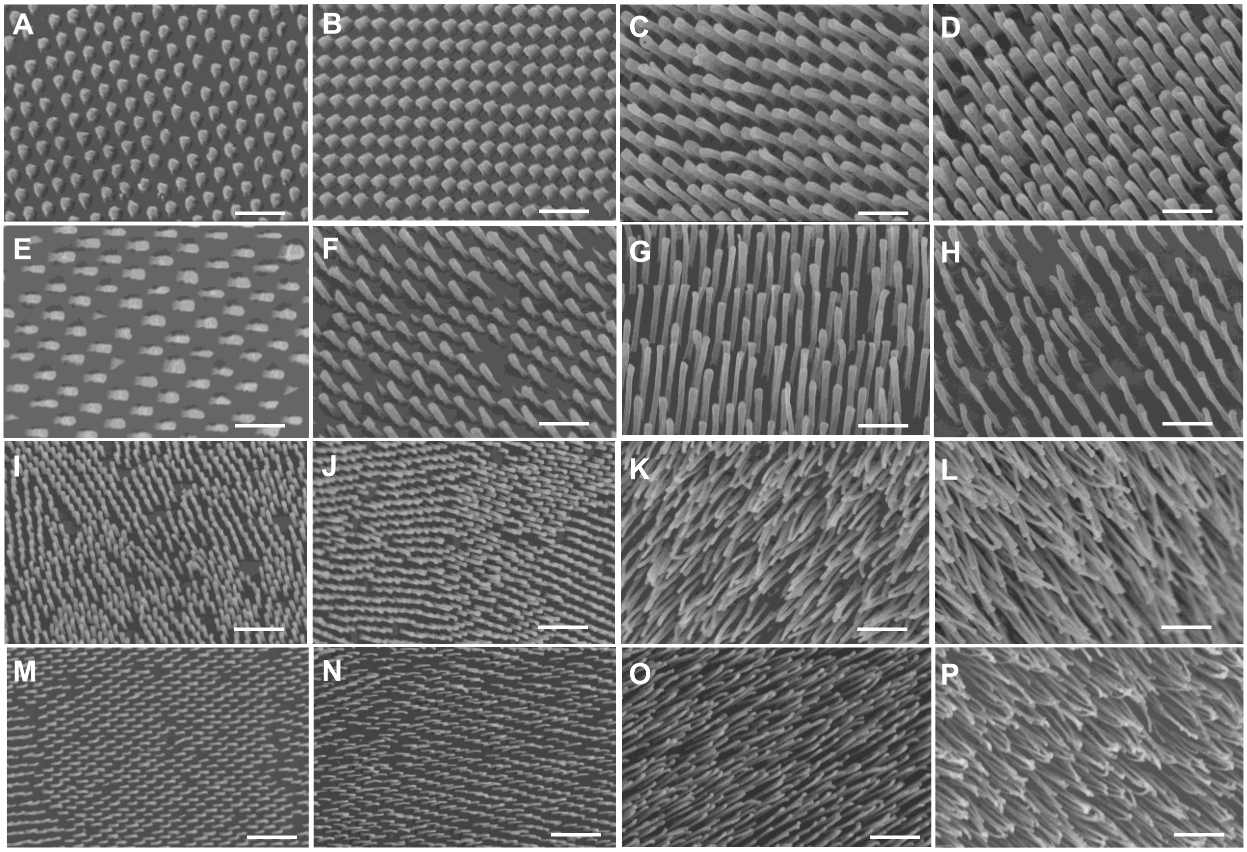

Fig. S2. **Tilted-view SEM micrographs showing the polyimide nanopillar arrays prepared with systematically varied geometries.** (**A-D**) 250 nm diameter, 500 nm pitch nanopillar arrays with the pillar heights varied from 200 nm (part **A**), 400 nm (part **B**), 800 nm (part **C**), to 1 μm (part **D**). (**E-H**) 100 nm diameter, 500 nm pitch nanopillar arrays with the pillar heights varied from 200 nm (part **E**), 400 nm (part **F**), 800 nm (part **G**), to 1 μm (part **H**). (**I-L**) 100 nm diameter, 240 nm pitch nanopillar arrays with the pillar heights varied from 200 nm (part **I**), 400 nm (part **J**), 800 nm (part **K**), to 1 μm (part **L**). (**M-P**) 50 nm diameter, 240 nm pitch nanopillar arrays with the pillar heights varied from 200 nm (part **M**), 400 nm (part **N**), 800 nm (part **O**), to 1 μm (part **P**). Scale bars: 1 μm.

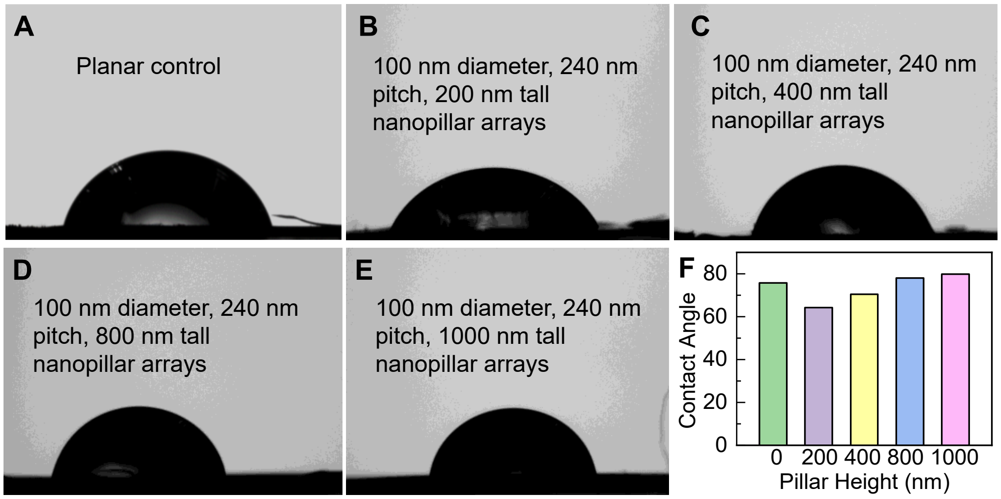

Fig. S3. **Impact of the nanopillar height on surface wettability with water.** (**A-E**) Static contact-angle images of a droplet of water on top of a planar Kapton foil (part **A**) and nanopillar arrays (part **B-E**). The pillar diameters were all 100 nm and pillar pitches were all 240 nm, while the pillar heights ranged from 200 nm (part **B**), 400 nm (part **C**), 800 nm (part **D**), to 1 μm (part **E**). (**F**) Measured contact angle of water on polyimide nanopillar arrays as a function of the pillar height.

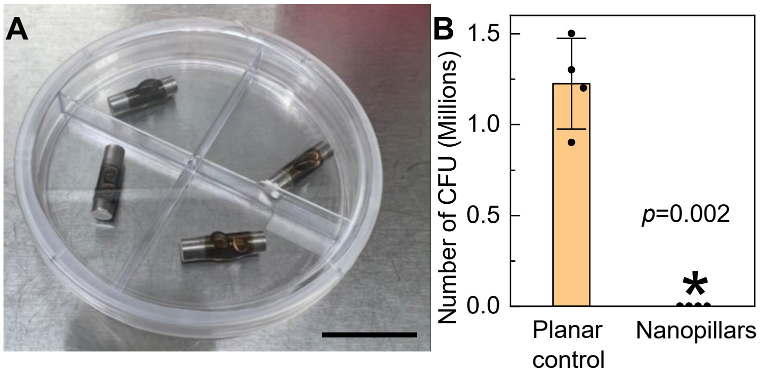

Fig. S4. **Impact of strain on the bactericidal efficacy of biomimetic physical antimicrobial nanopillar arrays.** (**A**) Optical picture showing the incubation of bacterial suspensions (*E. coli* strain MG1655) on smart-coating foils coated on the surface of 5.5 mm diameter titanium rods, with ~1.75% tensile strain applied due to bending. (**B**) The number of CFUs in the suspension after 3-hour incubation with the planar control and nanopillar arrays, under ~1.75% tensile strain applied (N=4). Virtually all bacteria were killed by nanopillars, as with flat foils (**Fig. 3I**). p value for unpaired t-test with unequal variance is 0.002.

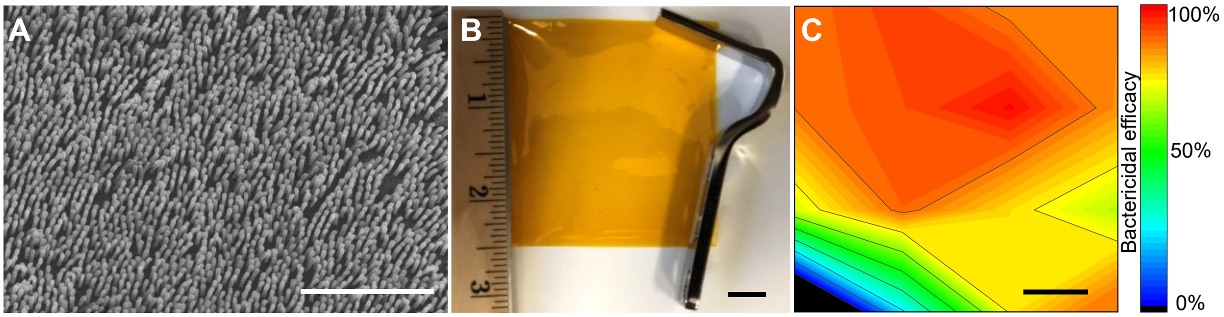

Fig. S5. **Robustness and large-area spatial uniformity of physical antimicrobial polymer nanopillar arrays.** (**A**) SEM image of the nanopillar arrays after Scotch-tape test. Scale bar: 2 µm. (**B-C**) Optical image of a 2.5" square polyimide foil featuring mechano-bactericidal nanopillar arrays on its top surface (part **B**), and the spatial mapping of the measured bactericidal efficacy, which is represented as the percentage of one million *E. coli* killed in suspension after 90 min incubation over each 1×1 cm^2 piece cut from the foil (part **C**). Scale bars: 1 cm.

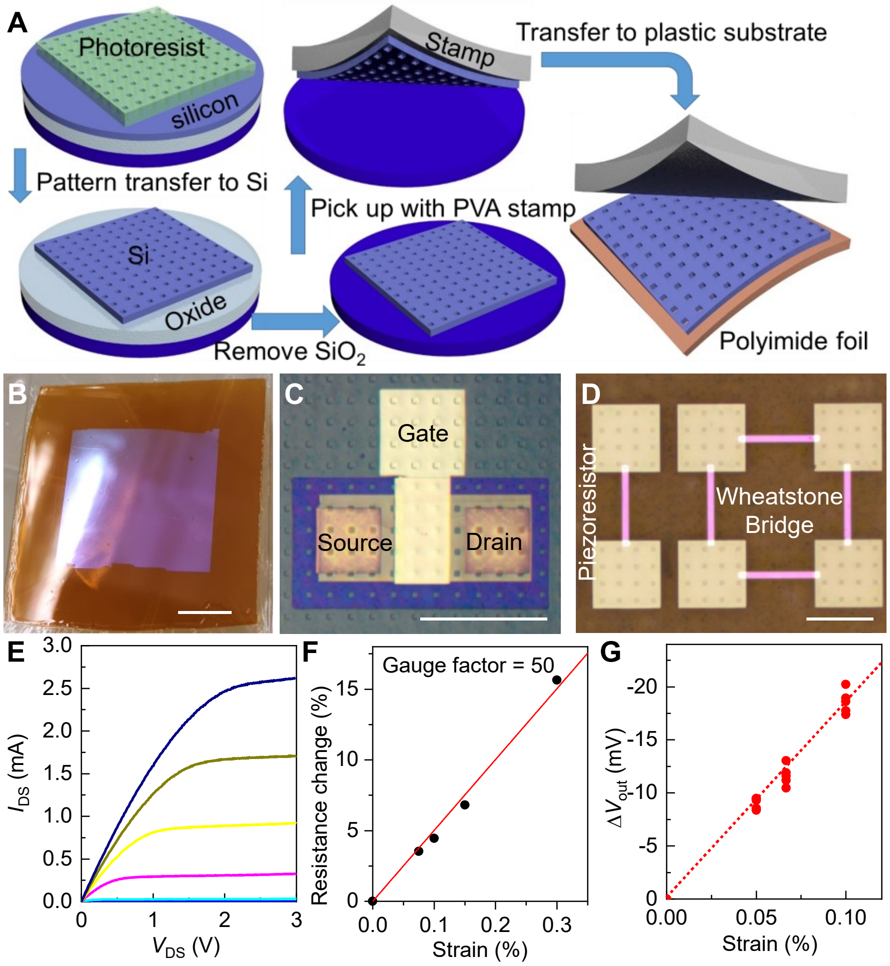

Fig. S6. Fabrication and characteristics of mechanically flexible selector transistors and strain sensors built on transfer-printed silicon nanomembranes. (**A**) Schematics showing the process to transfer-print single-crystalline silicon nanomembranes cultivated from silicon-on-insulator wafers to polymer foils. PVA: polyvinyl alcohol. (**B**) Optical image of a large-area silicon nanomembrane transferred on a Kapton-foil substrate. Scale bar: 1 cm. (**C-D**) Micrographs showing the completed transistors (part **C**) and piezoresistive strain sensors (part **D**). Scale bars: 200 μm. (**E**) Current-voltage characteristics of the selector transistor (channel width is 200 μm and channel length is 15 μm). The applied gate voltage decreases from 3 V to 0 V with a step of 0.5 V from top to bottom. (**F**) Relative change in resistance of a silicon-nanomembrane piezoresistor as a function of the applied longitudinal tensile strain, with the slope corresponding to a gauge factor of 50. (**G**) Voltage output (ΔV_{out}) from multiple Wheatstone-bridge strain gauges under tensile strain. The applied bias was 1 V.

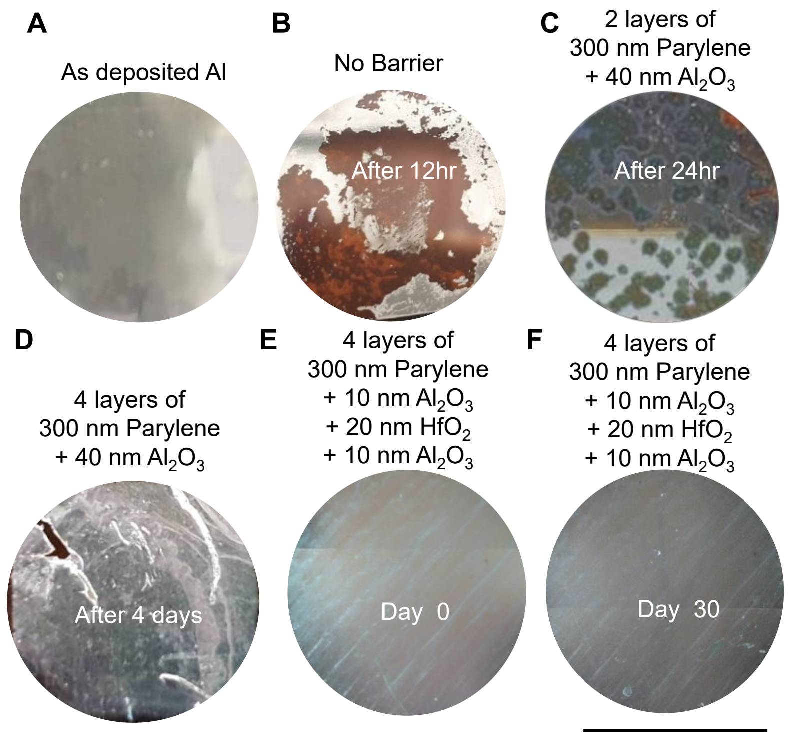

Fig. S7. **Biofluid barrier protecting electronics for chronic orthopedic implants.** (**A**) Optical image of a uniform layer of aluminum deposited on a Kapton foil as a convenient test vehicle to examine the barrier properties. (**B**) Defects in the aluminum film created by the reaction $2Al+6H_2O \rightarrow 2Al(OH)_3+3H_2$ after continuous immersion in hot PBS for 12 hours without passivation. (**C-D**) Optical images of the aluminum films passivated with two (part **C**) and four (part **D**) layers of parylene/Al_2O_3 stacks after immersion in hot PBS for one and four days, respectively. (**E-F**) Defect-free aluminum film passivated with four layers of the parylene/Al_2O_3/HfO_2/Al_2O_3 stacks before (part **E**) and after (part **F**) immersion in hot PBS for 30 days. Visible defects start to emerge afterwards. Scale bar: 5 mm.

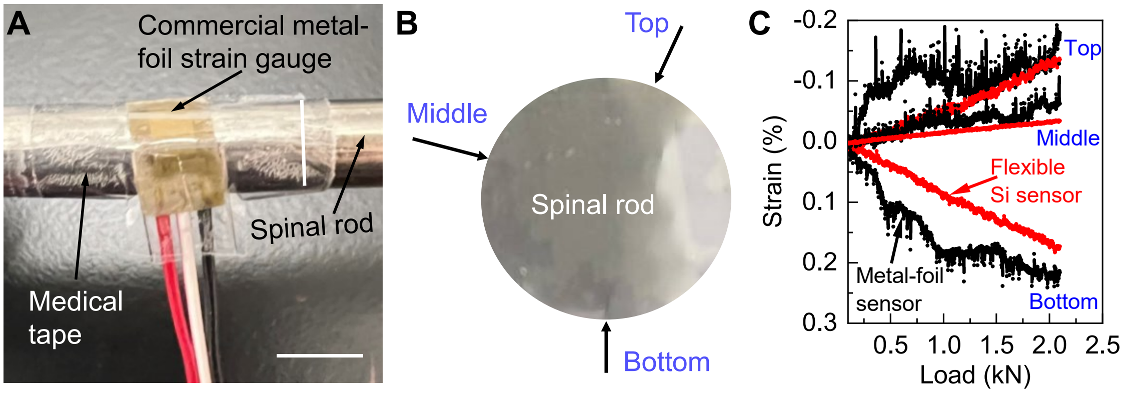

Fig. S8. **Benchmark the measurements performed using the commercial metal-foil strain gauges and the flexible silicon piezoresistive sensor arrays.** (**A**) Optical image showing a commercial metal-foil sensor applied on the surface of a spinal rod. Scale bar: 5 mm. (**B**) Schematic showing the three different locations where the metal-foil gauge was applied, corresponding with the positions of the top, middle, and bottom rows of the flexible silicon strain sensors in their multiplexed array. (**C**) Comparison of the local strain as a function of the load applied, as measured by the commercial metal-foil strain gauges (black) and the flexible single-crystalline silicon piezoresistive sensor array (red).

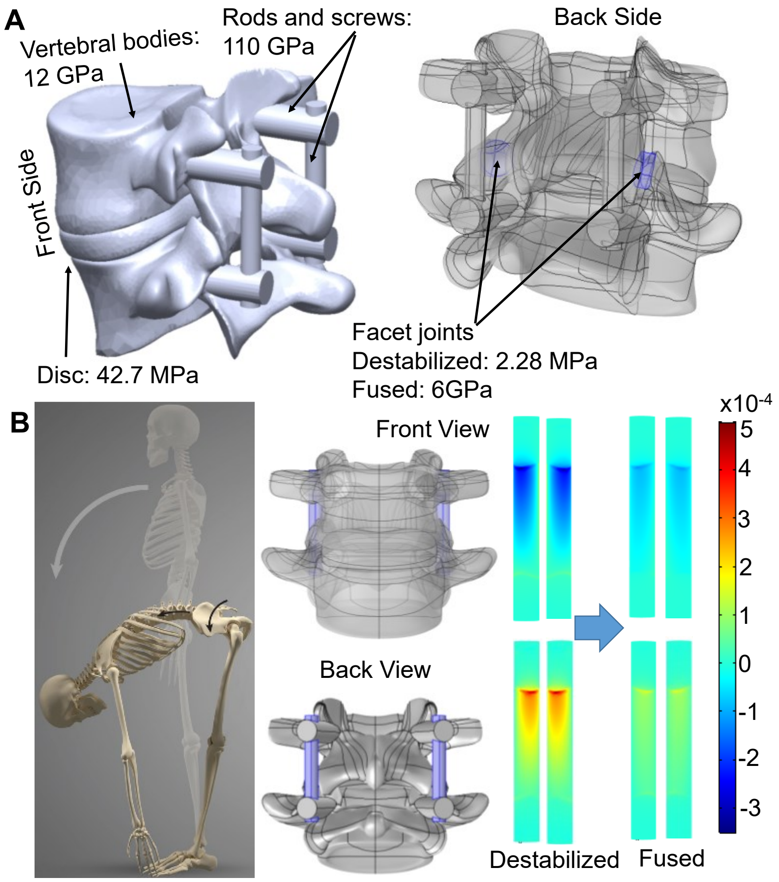

Fig. S9. **Biomechanical modeling showing the strain modulation on the spinal rod after fusion.** (**A**) Front (left part) and back (right part) side views of the model based on computed-tomography scan of a lumber spine segment. The Young's moduli of the vertebral bodies, disc, facet joints, rods, and screws were all extracted from literature and labeled. The modulus of the facet joints is assumed to reduce by 10 times from their typical value of 22.8 MPa after destabilization, and restored to 6 GPa, after early- to middle-stage fusion. (**B**) Simulated compressive and tensile strain distributions along the spinal rods before and after bony fusion in simulated flexion motion.

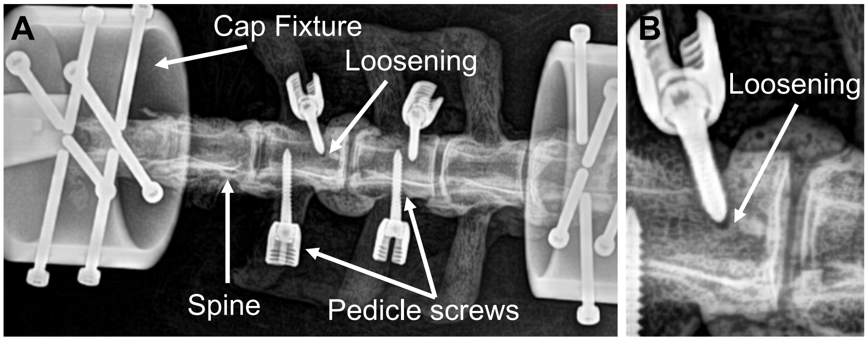

Fig. S10. **Radiological imaging of the pedicle-screw loosening.** (**A**) X-ray image showing the spine specimen with the pedicle screws and the cap fixtures attached**.** (**B**) Magnified view showing the loosening and extraction of one pedicle screw.

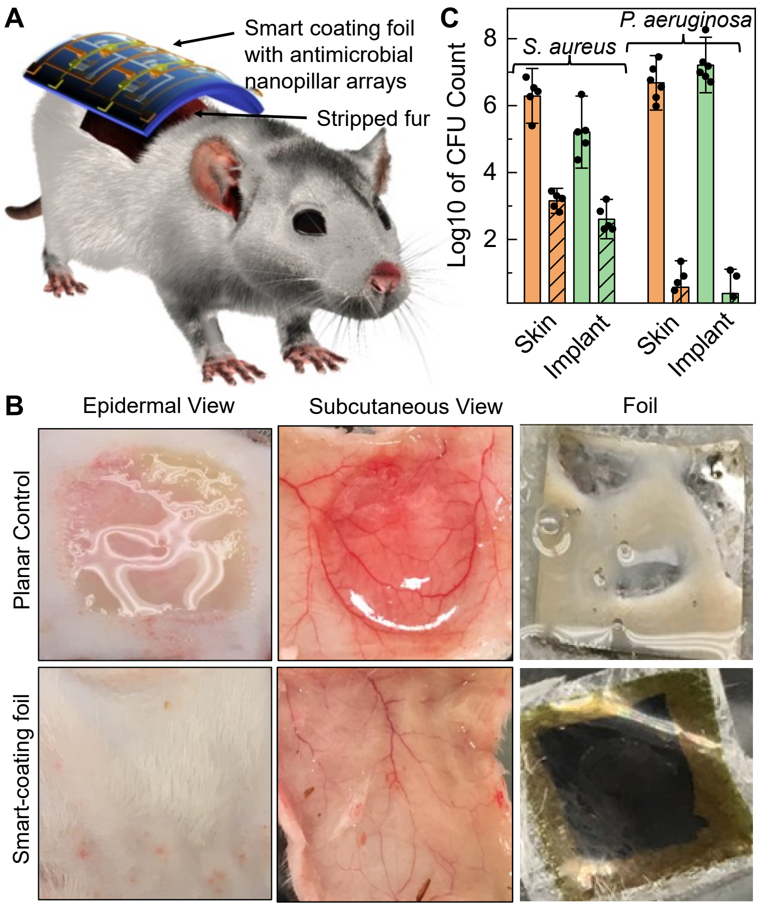

Fig. S11. Antimicrobial performance of the smart-coating foils authenticated in the superficial skin-infection model. (**A**) Schematic showing the mice superficial skin-infection model (7–8-week-old CD-1 mice, 5 per cohort, equal males and females), where the skin barrier was disrupted by the partial removal of the epidermal layer with tape stripping using Tensoplast, followed by the application of the foils inoculated with pathogenic bacteria using surgical tape. (**B**) Optical images showing the epidermal and subcutaneous tissues, together with the planar controls and the smart-coating foils applied after 3 days, both challenged with 5×10^6 CFUs of *P. aeruginosa* (278532). Compared to the planar controls, the smart-coating foils with biomimetic mechano-bactericidal nanopillar arrays can effectively prevent the superficial infection as evident from the absence of pus and hemorrhage. (**C**) Comparison of the *S. aureus* (N=5) and *P. aeruginosa* (N=6) burdens in the planar controls (green solid bars), the smart-coating foils featuring antimicrobial nanopillar arrays (green diagonal hatched bars), and their surrounding skin tissues (orange bars). p-values for unpaired t-test between the planar controls and smart-coating foils are all less than 0.0004.

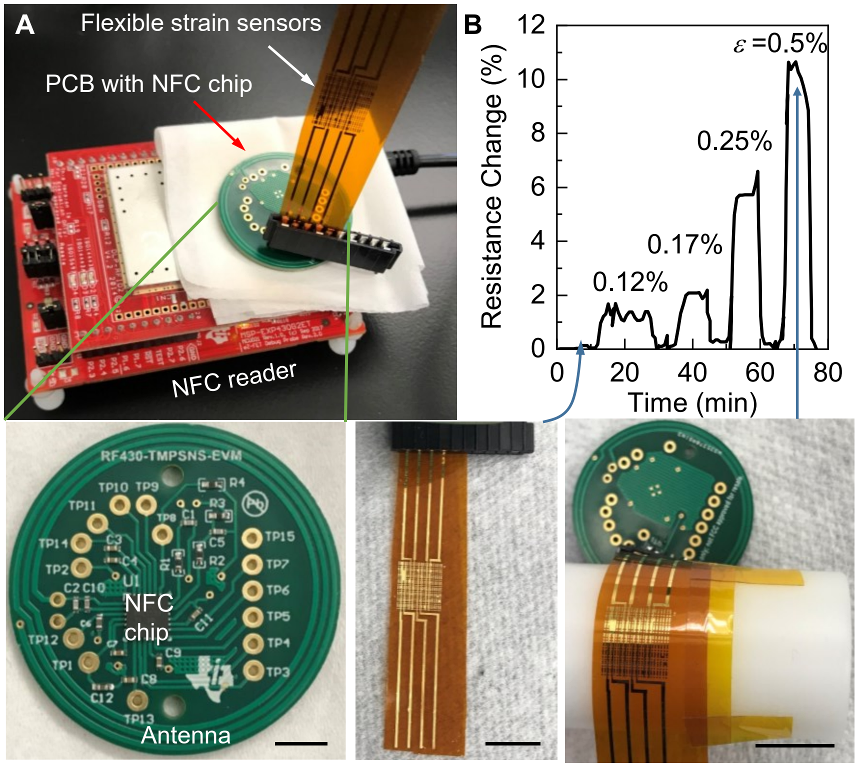

Fig. S12. Preliminary telemetry system for the smart-coating foils. (**A**) Optical image showing the flexible piezoresistive strain sensors, built on single-crystalline silicon nanomembrane transfer-printed onto a polyimide substrate, connected to a printed circuit board (PCB) containing antenna and a near-field communication (NFC) chip (RF430FRL152H, Texas Instrument). (**B**) Resistance change measured with the NFC telemetry system under different tensile strain applied by wrapping the flexible sensor on cylinders with different radii. Scale bars: 5 mm.

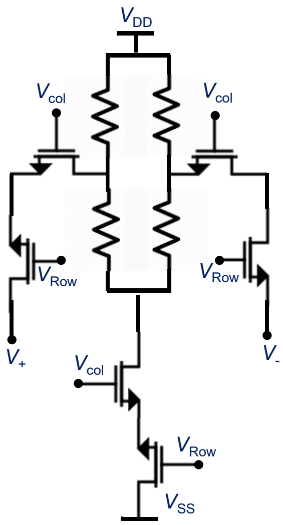

Fig. S13. Circuit diagram of strain-sensing pixels incorporating additional multiplexing transistors to turn off unselected pixels for lower energy consumption.

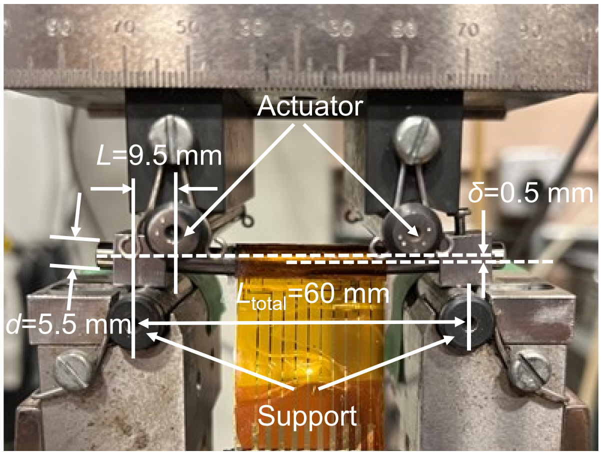

Fig. S14. **Deflection of the spinal rod in the four-point-bending test.** Applied load was 2 kN.

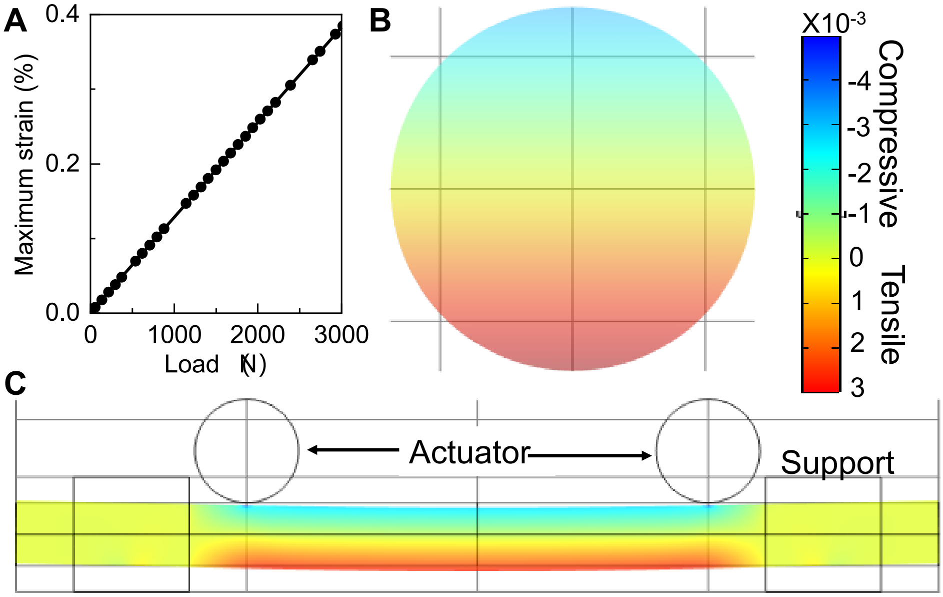

Fig. S15. **Finite-element simulation of strain on a spinal rod in four-point-bending test.** (**A**) Simulated maximum tensile strain as a function of the force load applied. (**B**) Simulated cross-sectional strain tensor distribution at the middle position along the spinal rod under 2 kN load applied. (**C**) Side view of the simulated deformation of the spinal rod and the corresponding strain tensor distribution.

REFERENCES AND NOTES

1. L. E. Bayliss, D. Culliford, A. P. Monk, S. Glyn-Jones, D. Prieto-Alhambra, A. Judge, C. Cooper, A. J. Carr, N. K. Arden, D. J. Beard, A. J. Price, The effect of patient age at intervention on risk of implant revision after total replacement of the hip or knee: A population-based cohort study. *Lancet* **389**, 1424–1430 (2017).

2. M.-J. Reisener, M. Pumberger, J. Shue, F. P. Girardi, A. P. Hughes, Trends in lumbar spinal fusion—A literature review. *J. Spine Surg.* **6**, 752–761 (2020).

3. B. H. Kapadia, R. A. Berg, J. A. Daley, J. Fritz, A. Bhave, M. A. Mont, Periprosthetic joint infection. *Lancet* **387**, 386–394 (2016).

4. E. Lenguerrand, M. R. Whitehouse, A. D. Beswick, S. K. Kunutsor, P. Foguet, M. Porter, A. W. Blom; National Joint Registry for England, Wales, Northern Ireland and the Isle of Man, Risk factors associated with revision for prosthetic joint infection following knee replacement: An observational cohort study from England and Wales. *Lancet Infect. Dis.* **19**, 589–600 (2019).

5. J. Zhou, R. Wang, X. Huo, W. Xiong, L. Kang, Y. Xue, Incidence of surgical site infection after spine surgery: A systematic review and meta-analysis. *Spine* **45**, 208–216 (2020).

6. R. O. Darouiche, Treatment of infections associated with surgical implants. *N. Engl. J. Med.* **350**, 1422–1429 (2004).

7. D. Hedequist, A. Haugen, T. Hresko, J. Emans, Failure of attempted implant retention in spinal deformity delayed surgical site infections. *Spine* **34**, 60–64 (2009).

8. D. R. Osmon, E. F. Berbari, A. R. Berendt, D. Lew, W. Zimmerli, J. M. Steckelberg, N. Rao, A. Hanssen, W. R. Wilson; Infectious Diseases Society of America, Diagnosis and management of prosthetic joint infection: Clinical practice guidelines by the infectious diseases society of America. *Clin. Infect. Dis.* **56**, e1–e25 (2012).

9. C. M. Magin, S. P. Cooper, A. B. Brennan, Non-toxic antifouling strategies. *Mater. Today* **13**, 36–44 (2010).

10. K. G. Neoh, E. T. Kang, Combating bacterial colonization on metals via polymer coatings: Relevance to marine and medical applications. *ACS Appl. Mater. Interfaces* **3**, 2808–2819 (2011).

11. K. Chae, W. Y. Jang, K. Park, J. Lee, H. Kim, K. Lee, C. K. Lee, Y. Lee, S. H. Lee, J. Seo, Antibacterial infection and immune-evasive coating for orthopedic implants. *Sci. Adv.* **6**, eabb0025 (2020).

12. E. M. Hetrick, M. H. Schoenfisch, Reducing implant-related infections: Active release strategies. *Chem. Soc. Rev.* **35**, 780–789 (2006).

13. C. T. Johnson, J. A. Wroe, R. Agarwal, K. E. Martin, R. E. Guldberg, R. M. Donlan, L. F. Westblade, A. J. García, Hydrogel delivery of lysostaphin eliminates orthopedic implant infection by *Staphylococcus aureus* and supports fracture healing. *Proc. Natl. Acad. Sci. U.S.A.* **115**, E4960–E4969 (2018).

14. W. Xi, V. Hegde, S. D. Zoller, H. Y. Park, C. M. Hart, T. Kondo, C. D. Hamad, Y. Hu, A. H. Loftin, D. O. Johansen, Z. Burke, S. Clarkson, C. Ishmael, K. Hori, Z. Mamouei, H. Okawa, I. Nishimura, N. M. Bernthal, T. Segura, Point-of-care antimicrobial coating protects orthopaedic implants from bacterial challenge. *Nat. Commun.* **12**, 5473 (2021).

15. C. R. Arciola, D. Campoccia, L. Montanaro, Implant infections: Adhesion, biofilm formation and immune evasion. *Nat. Rev. Microbiol.* **16**, 397–409 (2018).

16. I. Levin-Reisman, I. Ronin, O. Gefen, I. Braniss, N. Shoresh, N. Q. Balaban, Antibiotic tolerance facilitates the evolution of resistance. *Science* **355**, 826–830 (2017).

17. E. P. Lesho, M. Laguio-Vila, The slow-motion catastrophe of antimicrobial resistance and practical interventions for all prescribers. *Mayo Clinic Proc.* **94**, 1040–1047 (2019).

18. A. M. VanHook, Antibiotic-induced inflammation. *Sci. Signal.* **15**, eade1683 (2022).

19. S. Kalghatgi, C. S. Spina, J. C. Costello, M. Liesa, J. R. Morones-Ramirez, S. Slomovic, A. Molina, O. S. Shirihai, J. J. Collins, Bactericidal antibiotics induce mitochondrial dysfunction and oxidative damage in mammalian cells. *Sci. Transl. Med.* **5**, 192ra185 (2013).

20. J. Marchant, When antibiotics turn toxic. *Nature* **555**, 431–433 (2018).

21. R. A. Deyo, A. Nachemson, S. K. Mirza, Spinal-fusion surgery — The case for restraint. *N. Engl. J. Med.* **350**, 722–726 (2004).

22. P. Drees, A. Eckardt, R. E. Gay, S. Gay, L. C. Huber, Mechanisms of disease: Molecular insights into aseptic loosening of orthopedic implants. *Nat. Clin. Pract.* **3**, 165–171 (2007).

23. O. P. P. Temmerman, P. G. H. M. Raijmakers, J. Berkhof, E. F. L. David, R. Pijpers, M. A. Molenaar, O. S. Hoekstra, G. J. J. Teule, I. C. Heyligers, Diagnostic accuracy and interobserver variability of plain radiography, subtraction arthrography, nuclear arthrography, and bone scintigraphy in the assessment of aseptic femoral component loosening. *Arch. Orthop. Trauma Surg.* **126**, 316–323 (2006).

24. S. Sugiyama, M. Wullschleger, K. Wilson, R. Williams, B. Goss, Reliability of clinical measurement for assessing spinal fusion: An experimental sheep study. *Spine* **37**, 763–768 (2012).

25. E. H. Ledet, B. Liddle, K. Kradinova, S. Harper, Smart implants in orthopedic surgery, improving patient outcomes: A review. *Innov. Entrep. Health* **5**, 41–51 (2018).

26. L. Cai, A. Burton, D. A. Gonzales, K. A. Kasper, A. Azami, R. Peralta, M. Johnson, J. A. Bakall, E. Barron Villalobos, E. C. Ross, J. A. Szivek, D. S. Margolis, P. Gutruf, Osseosurface electronics—Thin, wireless, battery-free and multimodal musculoskeletal biointerfaces. *Nat. Commun.* **12**, 6707 (2021).

27. C. M. Boutry, Y. Kaizawa, B. C. Schroeder, A. Chortos, A. Legrand, Z. Wang, J. Chang, P. Fox, Z. Bao, A stretchable and biodegradable strain and pressure sensor for orthopaedic application. *Nat. Electron.* **1**, 314–321 (2018).

28. E. H. Ledet, D. D'Lima, P. Westerhoff, J. A. Szivek, R. A. Wachs, G. Bergmann, Implantable sensor technology: From research to clinical practice. *J. Am. Acad. Orthop. Surg.* **20**, 383–392 (2012).

29. D. P. Linklater, V. A. Baulin, S. Juodkazis, R. J. Crawford, P. Stoodley, E. P. Ivanova, Mechano-bactericidal actions of nanostructured surfaces. *Nat. Rev. Microbiol.* **19**, 8–22 (2021).

30. J. A. Rogers, M. G. Lagally, R. G. Nuzzo, Synthesis, assembly and applications of semiconductor nanomembranes. *Nature* **477**, 45–53 (2011).

31. A. Tripathy, P. Sen, B. Su, W. H. Briscoe, Natural and bioinspired nanostructured bactericidal surfaces. *Adv. Colloid Interface Sci.* **248**, 85–104 (2017).

32. A. Velic, J. Hasan, Z. Li, P. K. D. V. Yarlagadda, Mechanics of bacterial interaction and death on nanopatterned surfaces. *Biophys. J.* **120**, 217–231 (2021).

33. E. P. Ivanova, J. Hasan, H. K. Webb, G. Gervinskas, S. Juodkazis, V. K. Truong, A. H. F. Wu, R. N. Lamb, V. A. Baulin, G. S. Watson, J. A. Watson, D. E. Mainwaring, R. J. Crawford, Bactericidal activity of black silicon. *Nat. Commun.* **4**, 2838 (2013).

34. J. Jenkins, J. Mantell, C. Neal, A. Gholinia, P. Verkade, A. H. Nobbs, B. Su, Antibacterial effects of nanopillar surfaces are mediated by cell impedance, penetration and induction of oxidative stress. *Nat. Commun.* **11**, 1626 (2020).

35. M. Ganjian, K. Modaresifar, M. R. O. Ligeon, L. B. Kunkels, N. Tümer, L. Angeloni, C. W. Hagen, L. G. Otten, P. L. Hagedoorn, I. Apachitei, L. E. Fratila-Apachitei, A. A. Zadpoor, Nature helps: Toward bioinspired bactericidal nanopatterns. *Adv. Mater. Interfaces* **6**, 1900640 (2019).

36. K.-H. Tsui, X. Li, J. K. H. Tsoi, S. F. Leung, T. Lei, W. Y. Chak, C. Zhang, J. Chen, G. S. P. Cheung, Z. Fan, Low-cost, flexible, disinfectant-free and regular-array three-dimensional nanopyramid antibacterial films for clinical applications. *Nanoscale* **10**, 10436–10442 (2018).

37. S. Wu, F. Zuber, K. Maniura-Weber, J. Brugger, Q. Ren, Nanostructured surface topographies have an effect on bactericidal activity. *J. Nanobiotechnol.* **16**, 20 (2018).

38. M. Larrañaga-Altuna, A. Zabala, I. Llavori, O. Pearce, D. T. Nguyen, J. Caro, H. Mescheder, J. L. Endrino, G. Goel, W. N. Ayre, R. K. Seenivasagam, D. K. Tripathy, J. Armstrong, S. Goel, Bactericidal surfaces: An emerging 21st-century ultra-precision manufacturing and materials puzzle. *Appl. Phys. Rev.* **8**, 021303 (2021).

39. L. Crémet, S. Corvec, P. Bémer, L. Bret, C. Lebrun, B. Lesimple, A. F. Miegeville, A. Reynaud, D. Lepelletier, N. Caroff, Orthopaedic-implant infections by *Escherichia coli*: Molecular and phenotypic analysis of the causative strains. *J. Infect.* **64**, 169–175 (2012).

40. E. P. Ivanova, D. P. Linklater, M. Werner, V. A. Baulin, X. M. Xu, N. Vrancken, S. Rubanov, E. Hanssen, J. Wandiyanto, V. K. Truong, A. Elbourne, S. Maclaughlin, S. Juodkazis, R. J. Crawford, The multi-faceted mechano-bactericidal mechanism of nanostructured surfaces. *Proc. Natl. Acad. Sci. U.S.A.* **117**, 12598–12605 (2020).

41. D. Chandra, S. Yang, Stability of high-aspect-ratio micropillar arrays against adhesive and capillary forces. *Acc. Chem. Res.* **43**, 1080–1091 (2010).

42. C. E. Harper, C. J. Hernandez, Cell biomechanics and mechanobiology in bacteria: Challenges and opportunities. *APL Bioengineering* **4**, 021501 (2020).

43. S. M. Won, H. S. Kim, N. Lu, D. G. Kim, C. del Solar, T. Duenas, A. Ameen, J. A. Rogers, Piezoresistive strain sensors and multiplexed arrays using assemblies of single-crystalline silicon nanoribbons on plastic substrates. *IEEE Trans. Electron Devices* **58**, 4074–4078 (2011).

44. M. Park, M.-S. Kim, Y.-K. Park, J.-H. Ahn, Si membrane based tactile sensor with active matrix circuitry for artificial skin applications. *Appl. Phys. Lett.* **106**, 043502 (2015).

45. E. Song, R. Li, X. Jin, H. du, Y. Huang, J. Zhang, Y. Xia, H. Fang, Y. K. Lee, K. J. Yu, J. K. Chang, Y. Mei, M. A. Alam, Y. Huang, J. A. Rogers, Ultrathin trilayer assemblies as long-

lived barriers against water and ion penetration in flexible bioelectronic systems. *ACS Nano* **12**, 10317–10326 (2018).

46. W. Li, D. C. Rodger, E. Meng, J. D. Weiland, M. S. Humayun, Y. C. Tai, Wafer-level parylene packaging with integrated RF electronics for wireless retinal prostheses. *J. Microelectromech. Syst.* **19**, 735–742 (2010).

47. J. L. Collins, H. Moncada Hernandez, S. Habibi, C. E. Kendrick, Z. Wang, N. Bihari, P. L. Bergstrom, A. R. Minerick, Electrical and chemical characterizations of hafnium (IV) oxide films for biological lab-on-a-chip devices. *Thin Solid Films* **662**, 60–69 (2018).

48. D. S. Finch, T. Oreskovic, K. Ramadurai, C. F. Herrmann, S. M. George, R. L. Mahajan, Biocompatibility of atomic layer-deposited alumina thin films. *J. Biomed. Mater. Res. Part A* **87A**, 100–106 (2008).

49. T. J. Haley, K. Raymond, N. Komesu, H. C. Upham, The toxicologic and pharmacologic effects of hafnium salts. *Toxicol. Appl. Pharmacol.* **4**, 238–246 (1962).

50. T. Akamaru, N. Kawahara, S. Tim Yoon, A. Minamide, K. Su Kim, K. Tomita, W. C. Hutton, Adjacent segment motion after a simulated lumbar fusion in different sagittal alignments: A biomechanical analysis. *Spine* **28**, 1560–1566 (2003).

51. L. A. Ferrara, I. Gordon, M. Coquillette, R. Milks, A. J. Fleischman, S. Roy, V. K. Goel, E. C. Benzel, A preliminary biomechanical evaluation in a simulated spinal fusion model: Laboratory investigation. *J. Neurosurg. Spine* **7**, 542–548 (2007).

52. A. I. Stavrakis, J. A. Niska, J. H. Shahbazian, A. H. Loftin, R. I. Ramos, F. Billi, K. P. Francis, M. Otto, N. M. Bernthal, D. Z. Uslan, L. S. Miller, Combination prophylactic therapy with rifampin increases efficacy against an experimental *Staphylococcus epidermidis* subcutaneous implant-related infection. *Antimicrob. Agents Chemother.* **58**, 2377–2386 (2014).

53. N. M. Bernthal, A. I. Stavrakis, F. Billi, J. S. Cho, T. J. Kremen, S. I. Simon, A. L. Cheung, G. A. Finerman, J. R. Lieberman, J. S. Adams, L. S. Miller, A mouse model of post-

arthroplasty *Staphylococcus aureus* joint infection to evaluate in vivo the efficacy of antimicrobial implant coatings. *PLOS ONE* **5**, e12580 (2010).

54. E. Kugelberg, T. Norström, T. K. Petersen, T. Duvold, D. I. Andersson, D. Hughes, Establishment of a superficial skin infection model in mice by using *Staphylococcus aureus* and *Streptococcus pyogenes*. *Antimicrob. Agents Chemother.* **49**, 3435–3441 (2005).

55. S. Amin Yavari, S. M. Castenmiller, J. A. G. van Strijp, M. Croes, Combating implant infections: Shifting focus from bacteria to host. *Adv. Mater.* **32**, 2002962 (2020).

56. J. Jenkins, M. I. Ishak, M. Eales, A. Gholinia, S. Kulkarni, T. F. Keller, P. W. May, A. H. Nobbs, B. Su, Resolving physical interactions between bacteria and nanotopographies with focused ion beam scanning electron microscopy. *iScience* **24**, 102818 (2021).

57. M. I. Ishak, J. Jenkins, S. Kulkarni, T. F. Keller, W. H. Briscoe, A. H. Nobbs, B. Su, Insights into complex nanopillar-bacteria interactions: Roles of nanotopography and bacterial surface proteins. *J. Colloid Interface Sci.* **604**, 91–103 (2021).

58. D. Chandra, S. Yang, Capillary-force-induced clustering of micropillar arrays: Is it caused by isolated capillary bridges or by the lateral capillary meniscus interaction force? *Langmuir* **25**, 10430–10434 (2009).

59. N. Mitsuhashi, K. Fujieda, T. Tamura, S. Kawamoto, T. Takagi, K. Okubo, BodyParts3D: 3D structure database for anatomical concepts. *Nucleic Acids Res.* **37**, D782–D785 (2008).

60. I. El Bojairami, K. El-Monajjed, M. Driscoll, Development and validation of a timely and representative finite element human spine model for biomechanical simulations. *Sci. Rep.* **10**, 21519 (2020).

61. A. V. Subramani, P. E. Whitley, H. T. Garimella, R. H. Kraft, Fatigue damage prediction in the annulus of cervical spine intervertebral discs using finite element analysis. *Comput. Methods Biomech. Biomed. Eng.* **23**, 773–784 (2020).

62. B. Areias, S. C. Caetano, L. C. Sousa, M. Parente, R. N. Jorge, H. Sousa, J. M. Gonçalves, Numerical simulation of lateral and transforaminal lumbar interbody fusion, two minimally invasive surgical approaches. *Comput. Methods Biomech. Biomed. Eng.* **23**, 408–421 (2020).

63. M. Niinomi, Mechanical properties of biomedical titanium alloys. *Mater. Sci. Eng. A* **243**, 231–236 (1998).
